# The long non-coding RNA *Paupar* regulates the expression of both local and distal genes

**DOI:** 10.1002/embj.201386225

**Published:** 2014-02-01

**Authors:** Keith W Vance, Stephen N Sansom, Sheena Lee, Vladislava Chalei, Lesheng Kong, Sarah E Cooper, Peter L Oliver, Chris P Ponting

**Affiliations:** 1 MRC Functional Genomics Unit, University of OxfordOxford, UK; 2 CGAT, University of OxfordOxford, UK; 3 Department of Physiology, Anatomy and Genetics, University of OxfordOxford, UK; 4 Department of Biochemistry, University of OxfordOxford, UK

**Keywords:** CHART, CNS, lncRNA, PAX6, transcription

## Abstract

Although some long noncoding RNAs (lncRNAs) have been shown to regulate gene expression in *cis*, it remains unclear whether lncRNAs can directly regulate transcription in *trans* by interacting with chromatin genome-wide independently of their sites of synthesis. Here, we describe the genomically local and more distal functions of *Paupar*, a vertebrate-conserved and central nervous system-expressed lncRNA transcribed from a locus upstream of the gene encoding the PAX6 transcription factor. Knockdown of *Paupar* disrupts the normal cell cycle profile of neuroblastoma cells and induces neural differentiation. *Paupar* acts in a transcript-dependent manner both locally, to regulate *Pax6*, as well as distally by binding and regulating genes on multiple chromosomes, in part through physical association with PAX6 protein. *Paupar* binding sites are enriched near promoters and can function as transcriptional regulatory elements whose activity is modulated by *Paupar* transcript levels. Our findings demonstrate that a lncRNA can function in *trans* at transcriptional regulatory elements distinct from its site of synthesis to control large-scale transcriptional programmes.

## Introduction

The resolution of two key questions would greatly improve our understanding of the functions of long noncoding RNAs (lncRNAs; ≥200 nucleotides). First, is it more often the RNA product or else the act of transcription that conveys lncRNA function? Second, is any given lncRNA more likely to control transcription locally (in the vicinity of its locus) or else more distally in the genome?

A number of lncRNAs have been shown to regulate transcription of neighbouring genes on the same chromosome in an apparent *cis*-acting mechanism (Lai *et al*, 2013; Melo *et al*, 2013; Wang *et al*, 2011). These lncRNAs appear to function near their site of synthesis, in either an RNA-dependent manner to mediate looping onto the promoter regions of their transcriptional targets, or by using RNA-independent mechanisms to locally alter chromatin status. By contrast, lncRNA transcripts have also been proposed to regulate gene expression in *trans*, without influencing transcription of their genomically neighbouring genes (Guttman *et al*, 2011; Hung *et al*, 2011). *Trans-*acting lncRNAs include p53-induced lncRNAs involved in mediating the DNA damage response (Huarte *et al*, 2010; Hung *et al*, 2011), lncRNAs transcribed from within the promoters of cell cycle genes (Hung *et al*, 2011), lncRNAs that function in the control of pluripotency and lineage differentiation (Guttman *et al*, 2011) and those that are regulators of dosage compensation (Chu *et al*, 2011; Simon *et al*, 2011). Other examples include *Evf-2* which binds and modulates the activity of the homeodomain containing transcription factor Dlx2 (Feng *et al*, 2006), and *Hotair*, a lncRNA transcribed from the *HoxC* locus, which regulates the activity of *HoxD* cluster genes in *trans* and interacts with chromatin at over 800 regions genome-wide (Chu *et al*, 2011; Rinn *et al*, 2007). LncRNAs therefore have the potential to interact with chromatin and specifically target multiple different loci genome-wide.

LncRNA loci that are transcribed in the developing mouse central nervous system (CNS) show a preference to be located adjacent to transcription factor genes and thus may regulate their transcription (Ponjavic *et al*, 2009). Here, we investigate the transcriptional function of a CNS expressed, unspliced, and chromatin-associated intergenic lncRNA termed *Paupar* that is divergently transcribed 8.5 kb upstream of *Pax6*. *Paupar* was prioritised for detailed experimental investigation from among those we catalogued previously (Ponjavic *et al*, 2009) owing to the atypical evolutionary conservation of its sequence and transcription and because of its physical proximity to the transcription factor *Pax6*.

*Pax6* is required for eye and diencephalon specification and is known to control progenitor cell potency, progenitor cell proliferation, neuronal cell sub-type specification and spatial patterning in a dosage-sensitive manner (Georgala *et al*, 2011; Hill *et al*, 1991; Sansom *et al*, 2009; Shaham *et al*, 2012). Heterozygous human *PAX6* mutations can result in aniridia and in a variety of structural brain abnormalities that closely resemble those seen in *Small eye* (*sey*) mice heterozygous for *Pax6* mutations (Georgala *et al*, 2011; Hingorani *et al*, 2012). The proximity of the *Paupar* gene to *Pax6* suggested to us that it may be involved in the spatiotemporal control of *Pax6* expression and hence that it may be important for nervous system development and neurological disease.

Our results demonstrate functions for *Paupar* in the control of growth and differentiation in neural cells. In addition to conveying these functions locally, via transcriptional regulation of *Pax6*, we unexpectedly discovered that *Paupar* also functions distally in *trans* to control neural gene expression on a large scale. We mapped genome-wide *Paupar* occupancy in N2A neuroblastoma cells and identified hundreds of genes that are both bound and regulated by *Paupar*. We discovered that the *Paupar* transcript physically associates with PAX6 protein and that *Paupar* and PAX6 co-occupy specific genomic binding sites. Our results also revealed that *Paupar* associates in *trans* with functional elements involved in transcriptional control and that the *Paupar* transcript can modulate these elements’ activity. Our data therefore demonstrate that a single lncRNA transcript can bind and regulate the activity of multiple transcriptional regulatory elements on different chromosomes distinct from its site of synthesis.

## Results

### Conserved *Paupar* genomic organisation and transcription

**Figure 1:**
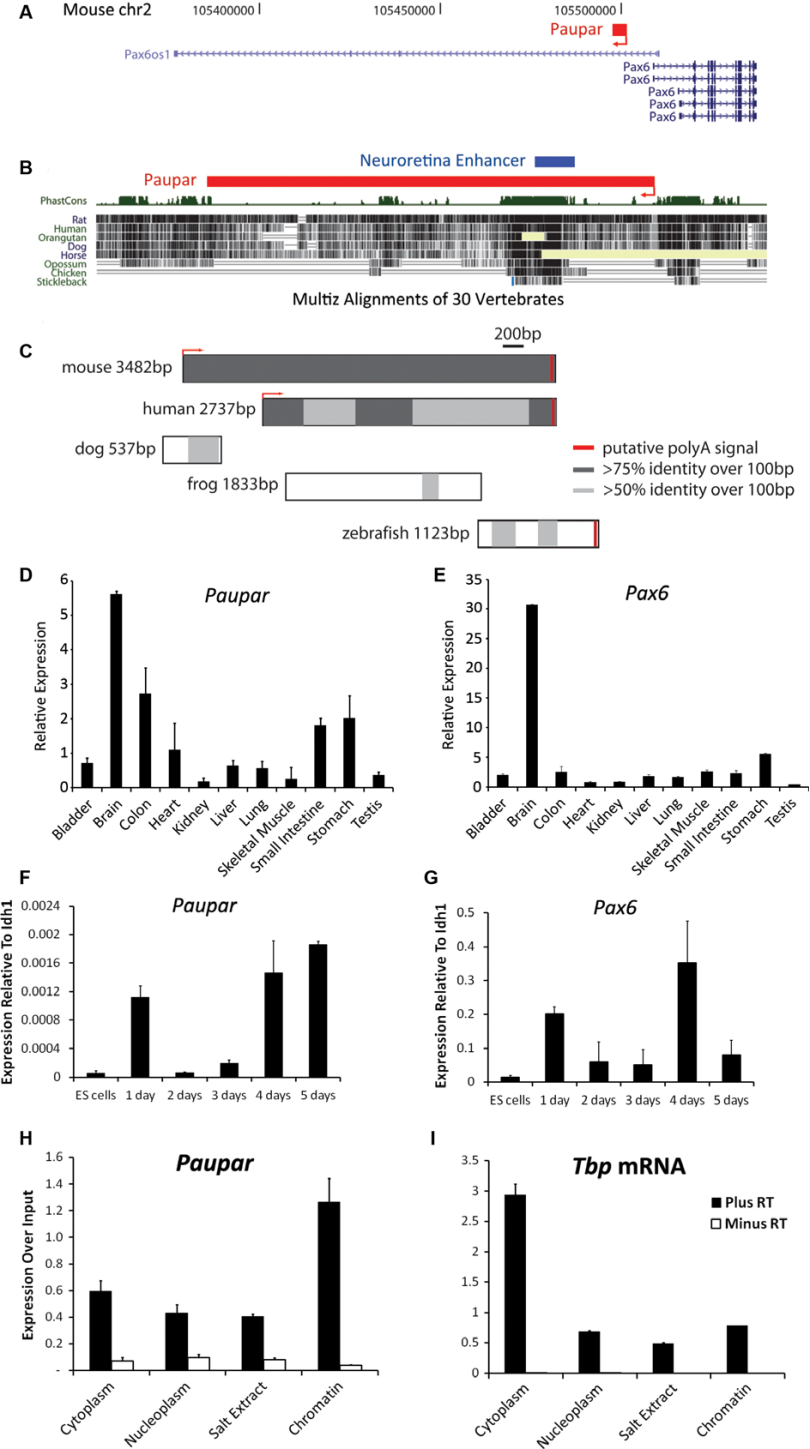
Conservation and expression of *Paupar*

RNA polymerase II-transcribed, CNS-expressed mouse lncRNAs tend to be evolutionarily constrained and to be preferentially located adjacent to transcriptional regulatory genes in the genome (Ponjavic *et al*, 2009, 2007). One of these lncRNAs (GenBank: AK032637), which we term *Paupar* (Pax6 Upstream Antisense RNA), is a single exon lncRNA transcribed from 8.5 kb upstream of the *Pax6* gene in mouse which lies entirely within the first intron of *Pax6os1*, a previously defined non-coding *Pax6* natural antisense transcript locus (Fig1A; Alfano *et al*, 2005). Rapid amplification of cDNA ends (RACE) experiments in mouse neuroblastoma cells extended AK032637 by approximately 700 bp at the 5′ end revealing mouse *Paupar* to be a 3.48 kb transcript (Fig1B). The *Paupar* locus contains two regions of high DNA sequence conservation across diverse vertebrates that unusually include fish and birds as well as mammals (Fig1B). The first of these regions lies just 5′ upstream of the *Paupar* transcriptional start site and is likely to contain this transcript's promoter sequence. The second lies within the transcribed sequence and encompasses both a previously identified *Pax6* neuroretina enhancer element (Plaza *et al*, 1999) and a region of the transcript that we predicted to contain a stem loop secondary structure (Ponjavic *et al*, 2009). The orthologous human transcript, transcribed from 8.6 kb upstream of the human *PAX6* gene, was identified in foetal brain using RT-PCR and RACE and shows three regions of high sequence identity to its mouse orthologue (Fig1C). *Paupar* transcripts are known from dog, as well as from more distantly related vertebrates, frog and zebrafish (Fig1C). *Paupar* therefore is unusual in exhibiting higher degrees of sequence and transcriptional conservation than most lncRNA loci (Cabili *et al*, 2011; Marques & Ponting, 2009; Ulitsky *et al*, 2011).

A Schematic illustration of the mouse *Pax6* genomic territory displaying coding and non-coding transcript structures (NCBI37/mm9).

B A detailed view of the mouse *Paupar* locus (red) indicating regions of vertebrate DNA sequence conservation and the location of sequence (blue) that, in human and quail, is a *Pax6* neuroretina enhancer (Plaza *et al*, 1999).

C Conservation and relative sizes of identified *Paupar* transcripts in vertebrates. For human and mouse *Paupar*, transcript start sites (arrows) and transcript ends were confirmed by RACE (primer sequences in Supplementary Table 1). The identified orthologous ESTs from dog (DN871729), frog (CX414799, DN054151 and DN054152), and zebrafish (CT684153 and CT684154) are unlikely to represent full-length transcripts. Each of these *Paupar* orthologues displays conserved genomic location and transcriptional orientation relative to *Pax6*.

D, E *Paupar* is a brain-expressed lncRNA. *Paupar* (D) and *Pax6* (E) expression levels were measured across a panel of adult mouse tissues using quantitative RT-PCR (qRT-PCR). Results are presented relative to the average value of *Gapdh* and *Tbp* reference genes. Mean values ± standard error (s.e.) shown, *n* = 3 replicates.

F, G Similarly to *Pax6*, *Paupar* is up-regulated during neuronal differentiation of mouse ES cells. Neuronal differentiation of mouse ES cells was induced using RA. We determined the levels of *Paupar* (F) and *Pax6* (G) using qRT-PCR. Results are expressed relative to an *Idh1* control which does not change significantly during differentiation. Mean ± s.e., *n* = 3.

H, I Paupar is a chromatin-associated transcript that functions to regulate *Pax6* expression. N2A cells were biochemically separated into cytoplasmic, nucleoplasm, 420 mM salt and chromatin fractions. The relative levels of *Paupar* (H) and a control mRNA (*Tbp*) (I) in each fraction were determined by qRT-PCR. Mean values ± s.e. of three independent experiments. RT, reverse transcriptase.

### *Paupar* transcript is chromatin associated and co-expressed with *Pax6* in the neural lineages

To begin our investigation of *Paupar* function we first characterised its expression profile and sub-cellular localisation. We found that mouse *Paupar* is most highly expressed in the adult brain (Fig1D) and shows a clear correspondence in expression profile with *Pax6* (Fig1E). Notably, *Paupar* is expressed in the developing cerebellum in both the internal and external granular layers, where *Pax6* is also strongly expressed (Supplementary Fig S1A). Given the apparent spatial co-expression of *Paupar* and *Pax6*, we then asked whether their expression is temporally coordinated during retinoic acid (RA)-induced differentiation of mouse E14 embryonic stem (ES) cells. While *Paupar* expression is undetectable in self-renewing ES cells, it is rapidly and transiently up-regulated after 1 day of RA treatment before increasing again at 4 days (Fig1F), a profile similar to that observed for *Pax6* (Fig1G). Mouse neuro 2A (N2A) neuroblastoma cells express both *Paupar* (at an average level of approximately 15 copies per cell [Supplementary Fig S1B]) and *Pax6*, but not *Pax6OS1* (Supplementary Fig S1C), and are widely used as an *in vitro* model of neuronal differentiation. In these cells, we found *Paupar* RNA (Fig1H), but not a control mRNA (*Tbp*; Fig1I), to be nuclear-enriched and located mainly in the chromatin fraction, and noted that ENCODE data show human *Paupar* to be similarly present in the nucleus and chromatin (ENCODE Project Consortium, 2012). Together, these data suggest that *Paupar* may act locally to regulate *Pax6* expression or that it may regulate similar biological processes as *Pax6*.

### *Paupar* regulates neural gene expression

**Figure 2:**
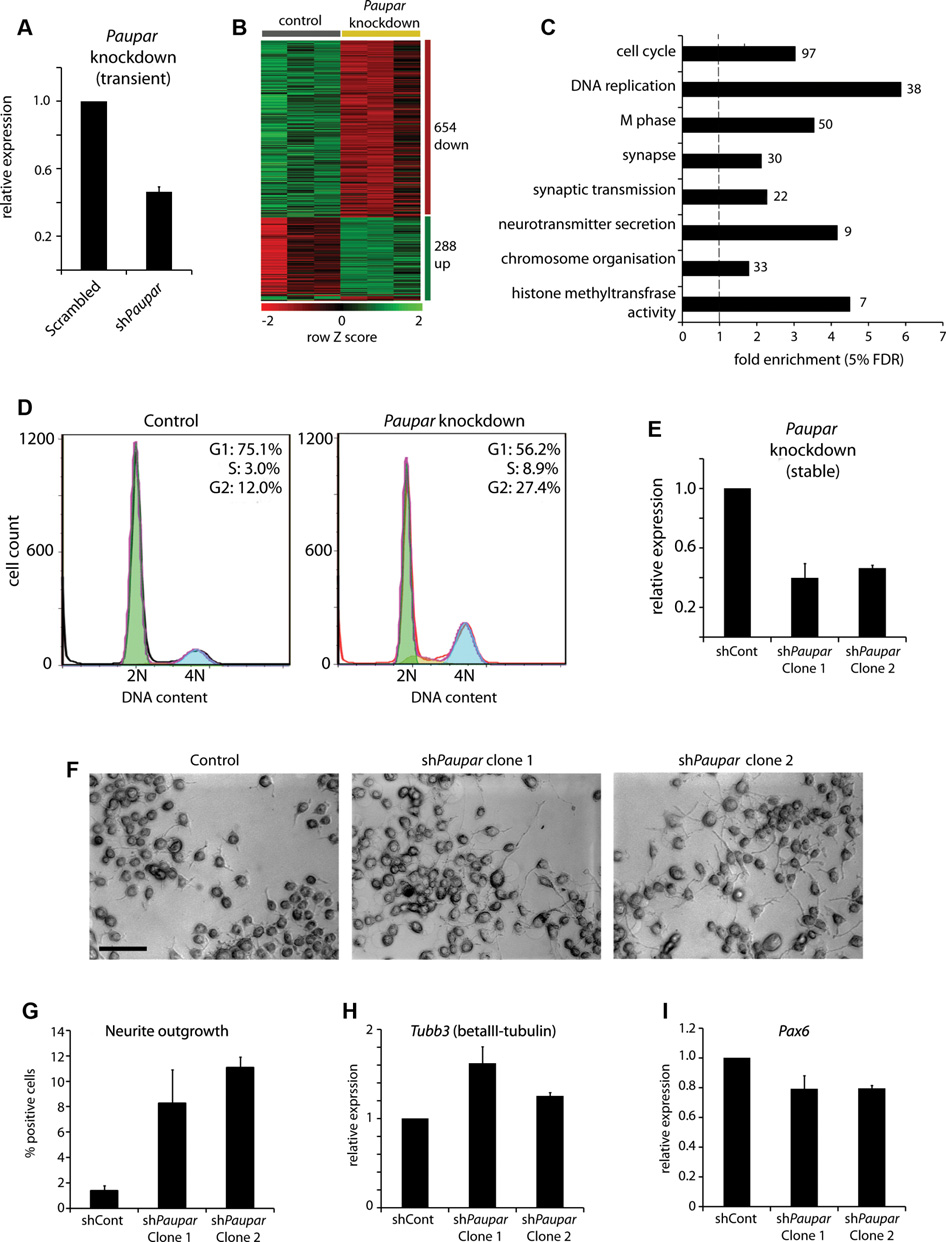
*Paupar* functions to regulate genes involved in cell cycle control and synaptic function

To investigate the functional importance of the *Paupar* transcript we performed transcriptome profiling of *Paupar* knockdown in N2A cells. We reduced *Paupar* expression by approximately 52%, using transient transfection of a *Paupar*-targeting shRNA expression vector (Fig2A), and verified that the chromatin-associated fraction of *Paupar* is depleted using this approach (Supplementary Fig S2A). *Paupar* knockdown resulted in statistically significant changes in the expression levels of 942 genes (False Discovery Rate [FDR] < 5%) compared to a non-targeting control (Supplementary Table 2); 654 (69%) of these genes were down-regulated and 288 (31%) were up-regulated (Fig2B).

A N2A cells were transfected with either a non-targeting control or a *Paupar*-targeting shRNA expression vector (sh408) and *Paupar* levels were determined by qRT-PCR 3 days later.

B *Paupar* knockdown induces statistically significant changes in the expression of 942 genes in N2A cells (5% FDR; Supplementary Table 2).

C Significant Gene Ontology annotation enrichments of *Paupar*-regulated genes (5% FDR, Supplementary Table 3).

D *Paupar* is important for normal S-phase progression and entry into mitosis. Mouse N2A cells were transfected with either a control or a *Paupar*-targeting shRNA expression vector. Three days later cells were fixed, stained with propidium iodide and the DNA content measured using flow cytometry.

E *Paupar* loss-of-function cell lines were generated by stable co-transfection of shRNA expression plasmids against either *Paupar* or a non-targeting control and a hygromycin expression vector for selection. qRT-PCR analysis confirms the generation of two clonal cell lines expressing reduced levels of *Paupar*. Mean values ± s.e.

F *Paupar* knockdown cells display increased neurite outgrowth. Control and *Paupar* knockdown cells were imaged using bright field microscopy. Scale bar, 50 μm.

G Quantification of neurite outgrowth. Cells with one or more neurites of length greater than twice the cell body diameter were scored as positive. Average values ± s.e., *n* = 3. A total of 100–200 cells were counted in each case.

H, I The relative levels of the neuronal differentiation marker *Tubb3* (H) and *Pax6* (I) were quantified in *Paupar* knockdown and control cells using qRT-PCR. Samples were normalised using *Gapdh* and are presented relative to expression in control cells (set arbitrarily to 1). Mean values ± s.e., *n* = 3.

*Paupar-*regulated genes are significantly enriched in those involved in cell cycle control, specifically DNA replication and mitosis, those playing a role in synaptic function, and those modifying chromatin and chromosome organisation (Fig2C, Supplementary Table 3). To validate the changes in expression observed from the microarrays, we performed qRT-PCR for 12 *Paupar*-regulated genes with two additional *Paupar*-targeting shRNA expression constructs (Supplementary Fig S2B). We observed consistent changes for all 12 genes and saw changes in expression commensurate with the strength of *Paupar* knockdown indicating that transcript level changes are specific and are not likely to result from off-target effects. Furthermore, *Paupar* overexpression induced dose-dependent changes in the expression of six out of eleven *Paupar*-regulated genes tested (Supplementary Fig S2C). The *Paupar* transcript therefore appears to function as a large-scale regulator of gene expression in neural cells.

### *Paupar* regulates neural growth and differentiation in N2A cells

We next investigated the role of *Paupar* in cell cycle control by assaying the effect of *Paupar* knock-down on the proliferation of N2A cells. *Paupar* knockdown cells accumulate in S and G2 phases (Fig2D) indicating that this transcript is necessary for normal passage through S phase and entry into mitosis. Taken together with the temporally regulated expression of *Paupar* during neural differentiation, these data indicate that *Paupar* may be important for the control of neural progenitor cell proliferation and differentiation.

To further investigate this hypothesis we generated stable *Paupar* loss-of-function N2A cell lines and analysed the role of the *Paupar* transcript in neural differentiation. We isolated and expanded two independent clones in which *Paupar* levels had been reduced by 50–60% (Fig2E). Strikingly, the *Paupar* knockdown clones showed a clear increase in neurite outgrowth, a well-characterised feature of neuronal differentiation, compared to a non-targeting control line (Fig2F and G). Additionally, the *Paupar* knockdown clones also showed increased levels of the neuronal differentiation marker *Tubb3* (encoding tubulin beta-3 chain) and a moderate reduction in *Pax6* which is known to be down-regulated upon neuronal differentiation (Fig2H and I). Together, these results indicate that *Paupar* regulates gene expression programmes that control neural growth and differentiation, acting to maintain the self-renewal of N2A cells.

### *Paupar* and *Pax6* have both common and distinct transcriptional targets

**Figure 3:**
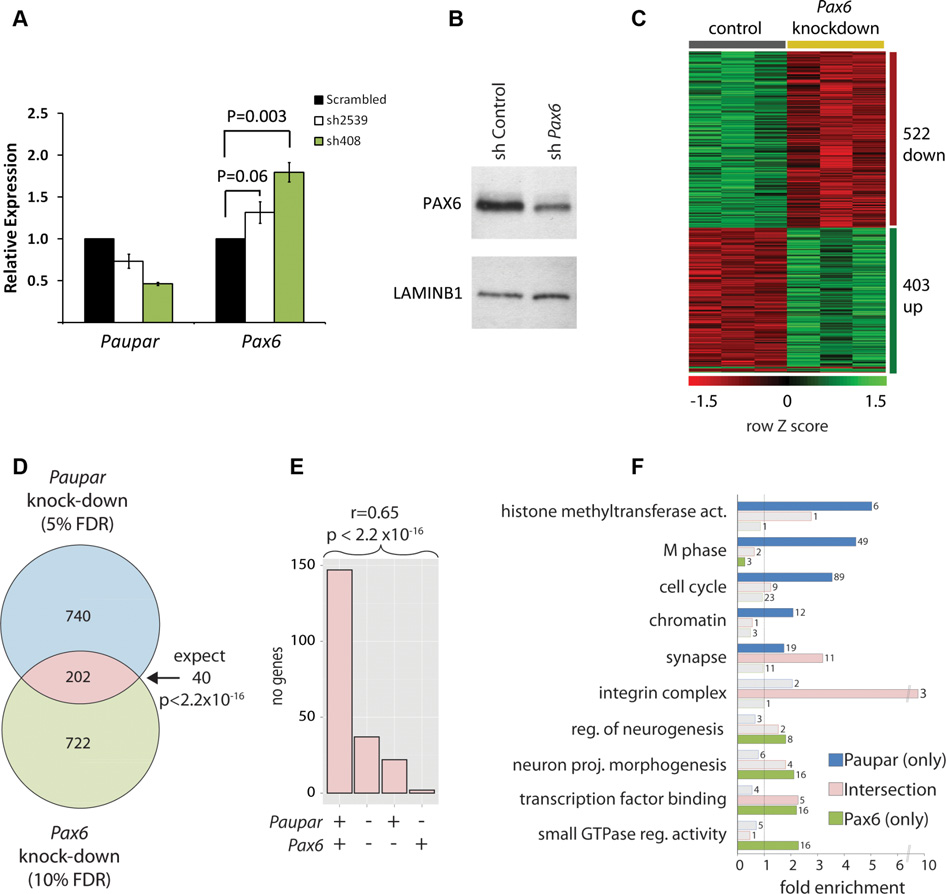
*Paupar* has both *Pax6*-dependent and -independent functions in transcriptional regulation

Given the known roles of *Pax6* in controlling neural stem cell fate, we next sought to further investigate the effect of *Paupar* RNA reduction on the expression of *Pax6*. While we observed a small decrease in *Pax6* transcript levels following stable *Paupar* knockdown (Fig2I), this finding cannot be interpreted unambiguously given that neural progenitor cells can down-regulate *Pax6* as they become neurogenic (Hsieh & Yang, 2009) and that *Pax6* is known to auto-regulate its own expression (Aota *et al*, 2003; Manuel *et al*, 2007). We therefore reduced *Paupar* levels with two distinct shRNAs transfected into N2A cells (Fig3A) and used qRT-PCR to measure acutely induced changes in *Pax6* expression. Transient reduction in *Paupar* RNA levels up-regulated *Pax6* in a dose-dependent manner: a maximum 54% reduction in *Paupar* levels resulted in an 80% increase in *Pax6* expression (Fig3A). These observations agreed with a small (1.2-fold), yet genome-wide non-significant, increase in *Pax6* expression detected on the *Paupar* knock-down microarrays.

*Paupar* knockdown leads to an increase in *Pax6* expression. N2A cells were transfected with two independent shRNA expression constructs targeting different regions of the *Paupar* transcript. The levels of *Paupar* and the adjacent *Pax6* gene were quantified using qRT-PCR 3 days later. Samples were normalised using *Gapdh* and the results are presented relative to a non-targeting control (set at 1). Mean values ± s.e., *n* = 4, one-tailed *t*-test, unequal variance.

Cells were transfected with either a non-targeting control or a *Pax6*-targeting shRNA expression vector and PAX6 protein levels were analysed by Western blotting 3 days later. Lamin B1 expression was used as a loading control.

*Pax6* knockdown resulted in statistically significant changes in the expression of 925 genes in N2A cells (10% FDR, Supplementary Table 2).

Intersection of *Pax6* and *Paupar* targets reveals a significant (Fisher's exact test) overlap approximately five times as large as expected by chance alone.

Target genes for both *Paupar* and *Pax6* show correlated expression, with the majority being positively regulated by both factors. +, positive dependency; −, negative dependency.

Enrichments of Gene Ontology categories in *Pax6* and *Paupar* target genes.

Given the ability of *Paupar* to regulate *Pax6* expression, we sought to determine the extent to which this could explain the *Paupar* transcriptional response. Reduction of PAX6 protein levels in N2A cells by approximately 70%, through the transient transfection of a *Pax6*-targeting shRNA expression vector (Fig3B), resulted in statistically significant expression level changes in 925 genes (FDR < 10%; Fig3C and Supplementary Table 2). Importantly, we noted no change in the levels of *Paupar* transcript upon *Pax6* knockdown (Supplementary Fig S3). Genes changing in expression, as expected from the role of PAX6 as a key neuro-developmental transcription factor, were enriched for genes involved in neurogenesis and transcription factor binding (Fig3F, Supplementary Table 3).

The set of genes showing significant expression changes in both *Paupar* and *Pax6* knock-downs was 5.1-fold greater than expected by chance (*P* < 2.2 × 10^−16^; Fig3D), consistent with *Paupar* regulating *Pax6* expression. A large majority of these genes showed positively correlated changes in expression for both *Paupar* and *Pax6* knock-down (Fig3E) indicating that while *Paupar* may repress *Pax6* transcription, *Paupar* RNA and PAX6 protein cooperate to coordinate the expression of a common set of target genes. However, notwithstanding the significant overlap between genes regulated by *Pax6* and *Paupar*, a large majority of *Paupar* responsive genes are not significantly altered by *Pax6* knockdown suggesting that *Paupar* may also possess *Pax6-*independent *trans*-acting functions. Notably, genes regulated by *Paupar* but not by *Pax6* are enriched for regulators of cell cycle control and chromatin organisation, while genes whose expression are controlled by both *Paupar* and *Pax6* include many with synaptic functions (Fig3F, Supplementary Table 3).

### Genome-wide binding profile of the *Paupar* lncRNA in N2A cells

**Figure 4:**
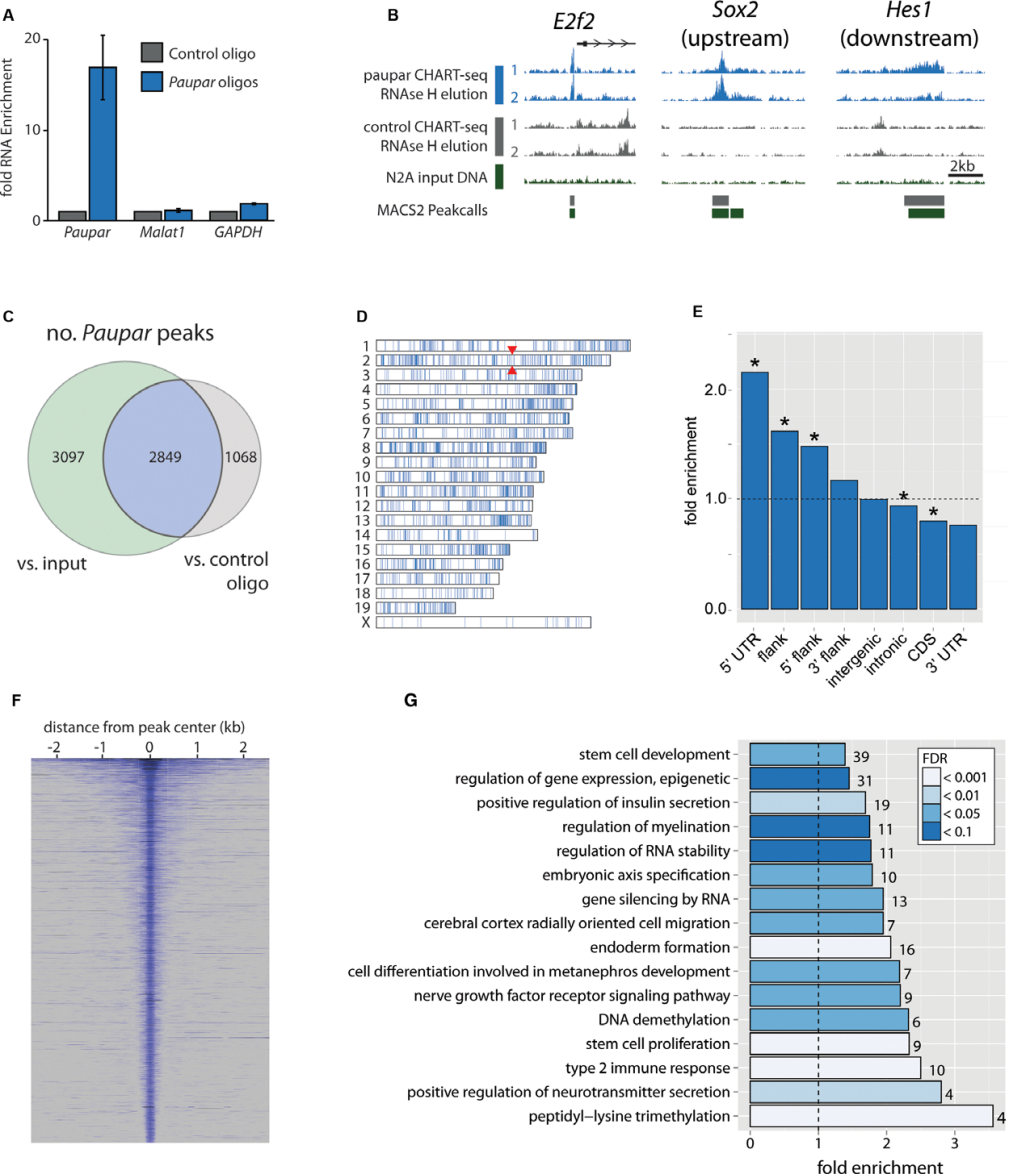
CHART-Seq analysis of *Paupar* genomic binding sites

We next investigated whether *Paupar* might function as a *trans*-acting transcriptional regulator by binding to genomic locations distal to its own locus. We first mapped the genome-wide binding profile of *Paupar* using the recently described Capture Hybridisation Analysis of RNA Targets (CHART)-Seq technique (Simon, 2013; Simon *et al*, 2011) in N2A cells. This approach uses anti-sense oligonucleotides to purify target lncRNAs and their associated chromatin complexes and thus identifies both direct and indirect genomic associations. We mapped accessible regions of the *Paupar* transcript based on RNase H sensitivity and designed four biotinylated DNA oligonucleotides, complementary to these regions (Supplementary Fig S4). For a control oligonucleotide, we used a sequence corresponding to *Escherichia coli LacZ* which is absent from the mouse genome. *Paupar* probes showed strong enrichment (17-fold) of the *Paupar* transcript compared to the *LacZ* control (Fig4A), and did not enrich for negative control transcripts, *Malat1*, a nuclear lncRNA, or *Gapdh* mRNA. As expected from physical association of a nascent transcript with its site of synthesis, we observed specific enrichment of *Paupar* at its DNA locus (Supplementary Fig S4).

A Specific enrichment of *Paupar* RNA using oligonucleotides complementary to accessible regions of *Paupar*, as determined by RNase H mapping (see Supplementary Fig S4), compared to the *LacZ* control. Mean value ± s.e., *n* = 4.

B Sequencing of *Paupar*-bound DNA (RNase H elution) reveals peaks of *Paupar* binding, including those at the promoter of *E2f2*, upstream of *Sox2* and downstream of *Hes1*.

C–E Peaks were called by comparing with sequences both from control CHART-seq experiments and from input DNA. Here we only consider the 2,849 peaks common to both comparisons (C, and Supplementary Table 4). *Paupar* peaks are broadly distributed across the mouse genome (D) but are particularly enriched in 5′ UTRs and gene promoters (E). Red arrowheads in (D) indicate the position of the *Paupar* locus. Asterisks in (E) indicate significance at 5% FDR (Benjamini-Hochberg).

F The width distribution of *Paupar* binding peaks.

G Representative categories from Gene Ontology analysis of genes associated with *Paupar* binding sites reveal enrichments for stem cell and neuronal categories amongst others (Supplementary Table 6).

Following the CHART-seq protocol, we used RNase H elution to recover genomic DNA associated with endogenous *Paupar* transcripts and genomic DNA associated with the control oligonucleotide, sequencing replicate samples using the Illumina HiSeq system. Using the paired-end peak caller MACS2 (Zhang *et al*, 2008), we identified *Paupar* binding sites in comparison both to DNA recovered using the control *LacZ* oligonucleotide and to input DNA from N2A cells. We discovered thousands of peaks across the genome, for example at the transcriptional start site (TSS) of *E2f2*, and at sites upstream of *Sox2* and downstream of *Hes1* (Fig4B) and defined *Paupar* binding sites as those peaks found in comparison to both input DNA and the control oligonucleotide samples (Fig4C). *Paupar* occupancy at nine candidate binding sites was validated using CHART-qPCR in two further independent experiments (Supplementary Fig S4).

These 2,849 candidate *Paupar* binding sites (Supplementary Table 4) are generally widely distributed across the genome, show a significant three-fold depletion on the X chromosome (*P* < 0.001 by genome-wide simulation accounting for mappability and GC biases (Heger *et al* (2013); Supplementary Table 5; Fig4D), and occur preferentially within the promoters and 5′ UTRs of protein-coding genes (Fig4E). Candidate *Paupar* binding sites range from narrower focal peaks of 200–1,000 bp, similar to those previously described for *Hotair* and *Terc* lncRNAs (Chu *et al*, 2011), to broader genomic regions of up to 9.5 kb (Fig4F).

We examined the sequence of the *Paupar* binding locations for clues as to its genomic targeting. Using a local alignment approach (see Materials and Methods), we did not find evidence for sequence complementarity between *Paupar* and its binding locations. However, *de novo* motif discovery (Supplementary Fig S5) identified a motif closely resembling a known PAX6 DNA binding motif in 9.2% of the top 500 scoring *Paupar* binding locations (Supplementary Fig S5C). Further analysis of *Paupar* CHART-Seq peaks for the presence of known vertebrate transcription factor motifs revealed enrichment of motifs for several neural transcription factors (Supplementary Table 7). Together, these results suggest that *Paupar* is not targeted to the genome through direct RNA-DNA interactions but that instead it interacts with PAX6 and other neural transcription factors to target specific genomic regions in a context-dependent manner.

### *Paupar* binding sites are associated with genes and regulatory regions of known stem cell and neural function

Using Gene Ontology assignments (Ashburner *et al*, 2000), we next sought to characterise the set of genes associated with *Paupar* binding locations (Fig4G, Supplementary Table 6). This analysis revealed enriched stem cell categories with peaks associated with many key stem cell genes such as *Sox2*, *Nanog*, *Hes1*, *Hes5*, *Rbpj* and *Lif*. We also found *Paupar* peaks to be enriched for neuronal gene categories as well as in genes whose products are important for the epigenetic regulation of gene expression. Intriguingly, *Paupar* binding sites are also enriched in genes associated with insulin secretion, a process in which *Pax6* has an established role (Gosmain *et al*, 2012).

**Figure 5:**
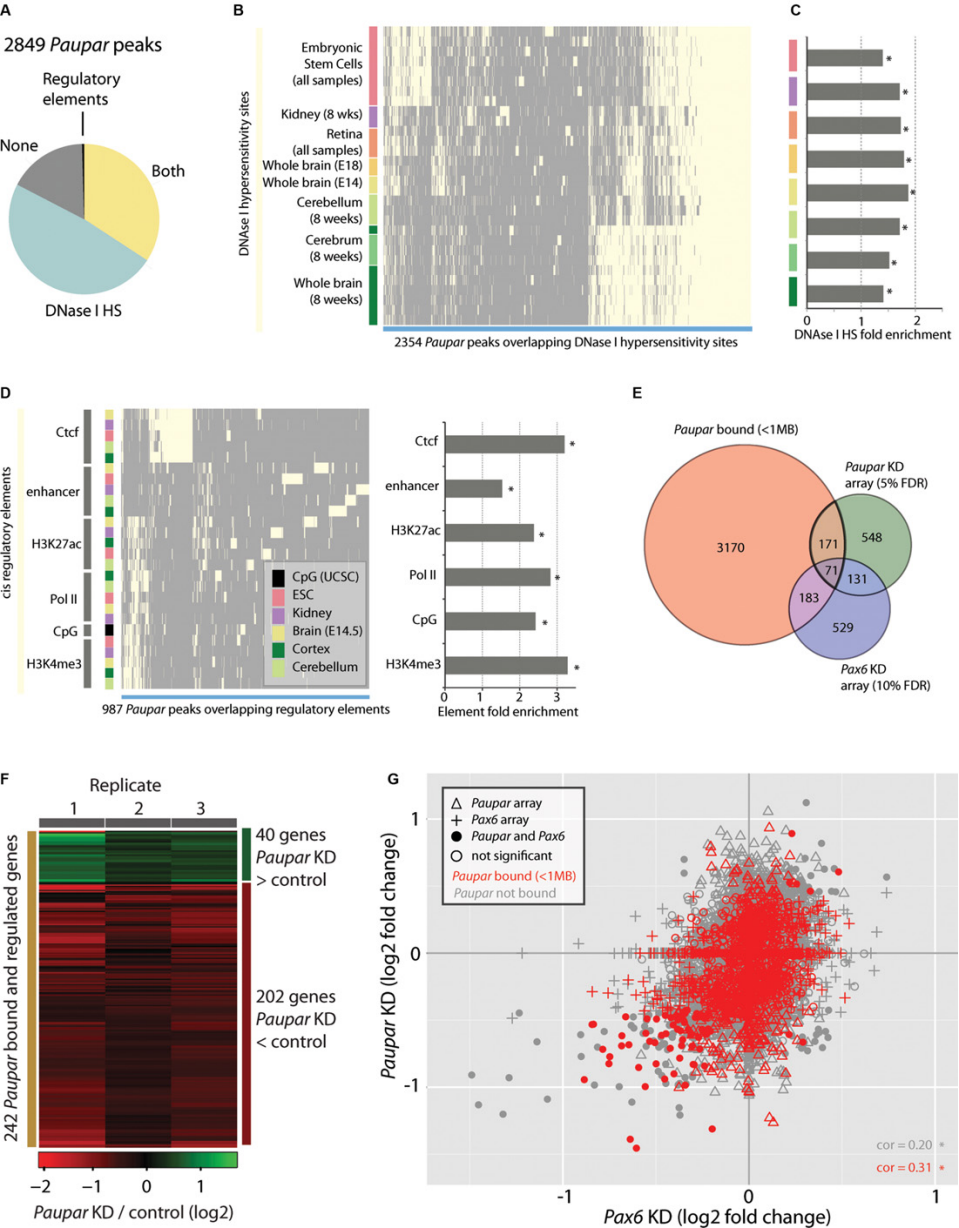
Association of *Paupar* binding sites with regulatory regions

To gain insight into the mechanism by which *Paupar* regulates gene expression, we examined its binding in relation to known functional regulatory regions. Firstly, we found that the majority of *Paupar* binding sites overlap DNase I hypersensitivity (HS) sites and *cis*-regulatory elements identified by the Mouse ENCODE Project (Shen *et al*, 2012; Fig5A, Supplementary Table 4). A large subset of binding sites are significantly associated with neuronal DNase I HS sites and a further subset are associated with DNase I HS sites found in embryonic stem cells (Fig5B and 5C). In agreement with the enrichment of *Paupar* binding sites at 5′ UTRs and promoters, we also observed a set of binding sites associated with features characteristic of transcriptional start sites including Pol II, predicted CpG islands, and tri-methylation of histone H3 at lysine 4 (H3K4me3; Fig5D). Additionally, we saw significant overlaps with Ctcf-binding regulatory elements and tissue-specific enhancers, defined using ratios of H3K4 mono- and tri-methylation (Shen *et al*, 2012).

The majority of *Paupar* binding sites overlap with DNase I HS sites and transcriptional regulatory elements as identified by the ENCODE project (selected tissues; Supplementary Table 5).

Hierarchical clustering of HS sites intersecting *Paupar* binding sites (light yellow) reveals large clusters of HS sites identified in neural tissues and embryonic stem cells that are bound by *Paupar* in N2A cells.

Fold enrichment of *Paupar* CHART-Seq-DNase I HS site associations.

Hierarchical clustering of *cis*-regulatory elements and *Paupar* peaks show groups of *Paupar* peaks associated with promoter-like features (H3K4me3, Pol II binding, CpG island predictions), peaks associated with Ctcf and peaks associated with tissue-specific enhancer elements.

The intersection of genes proximal (< 1 Mb) to *Paupar* peaks and genes changing expression upon *Paupar* (5% FDR) and *Pax6* (10% FDR) knockdown.

Heatmap displaying expression changes in the 242 *Paupar* bound and regulated genes from (E).

Analysis of *Paupar*-associated genes and changes in expression from *Paupar* and *Pax6* knockdown array experiments reveals a set of genes positively regulated by both *Paupar* and *Pax6* and directly bound by *Paupar* (red circles, lower left). Correlations (*cor*) are significant at *P* < 2 × 10^−16^.

Data information: Asterisks (C, D) indicate significance at 5% FDR (Benjamini-Hochberg).

### A subset of *Paupar*-regulated genes is also associated with *Paupar* binding

To investigate the functional consequences of *Paupar* binding we intersected the *Paupar* CHART-Seq peak-set with our microarray analysis of *Paupar* and *Pax6-*mediated gene expression changes. This identified 242 *Paupar*-bound and -regulated genes (5% FDR), representing likely direct transcriptional targets, and 254 *Paupar*-bound and *Pax6*-regulated genes (10% FDR; Fig5E). Hierarchical clustering of the 242 *Paupar-*bound and -regulated genes indicated that while *Paupar* predominantly activates the expression of target genes it can also function to repress certain loci (Fig5F). In accordance with this, genes associated with *Paupar* binding sites showed significantly higher expression in N2A cells than other genes (Supplementary Fig S6A, *P* < 2 × 10^−16^).

Notably, genes regulated by both *Paupar* and *Pax6* showed a small but significant enrichment for associated *Paupar* binding sites (Supplementary Fig S6B). To dissect the relationship between gene expression changes in the *Paupar* and *Pax6* knockdown experiments and the association of genes with *Paupar* binding further, we plotted these observations (Fig5G). First, we noted a moderate significant correlation (0.20, *P *<* *2.2 × 10^−16^) between all genes tested for differential expression in the two knockdown experiments, and that this correlation was stronger for genes also associated with *Paupar* binding sites (0.31, *P *<* *2.2 × 10^−16^). Secondly, we noted a distinct group of genes that are significantly positively regulated by both *Paupar* (5% FDR) and *Pax6* (10% FDR) and associated with *Paupar* binding (red circles, lower left Fig5G). In line with the observed intersection between genes regulated by *Paupar* and *Pax6* (Fig3), these findings suggest that *Paupar* and *Pax6* co-operate to regulate a set of common target genes (red circles, Fig5G) and further that *Paupar* can act independently of *Pax6* to directly regulate the expression of a separate set of genes (red triangles).

### *Paupar* and PAX6 co-occupy specific genomic binding sites

**Figure 6:**
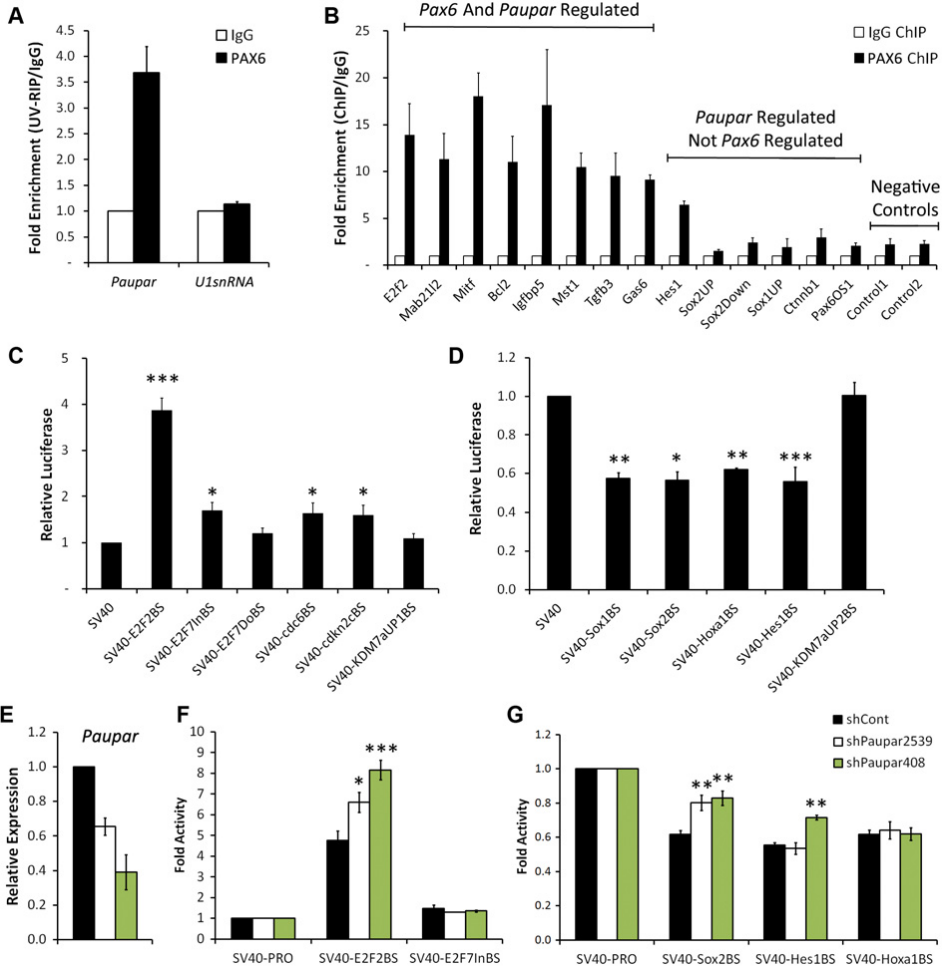
*Paupar* functions in *trans* to modulate the activity of neurodevelopmental gene transcriptional regulatory elements

The identification of 71 *Paupar* and *Pax6* co-regulated genes that are also bound by *Paupar* in their regulatory regions (Fig5E) together with the discovery of the known PAX6 DNA binding motif from *Paupar* bound sequences (Supplementary Fig S5) suggested a functional interaction between PAX6 and *Paupar*. To investigate this, we first tested for a physical association between PAX6 protein and the *Paupar* transcript using UV cross-linking RNA immunoprecipitation (UV-RIP) in N2A cells and detected a fourfold enrichment of *Paupar* using an anti-PAX6 antibody compared to an isotype control (Fig6A). The *Paupar*-PAX6 interaction appears to be specific as we observed no enrichment of a negative control *U1* snRNA transcript.

A *Paupar* interacts with PAX6 protein in N2A cells. Nuclear extracts were prepared from UV cross-linked cells and immuno-precipitated using either anti-PAX6 or control IgG antibodies. Associated RNAs were purified and the levels of *Paupar* and control *U1 snRNA* detected in each UV-RIP using qRT-PCR. Results are expressed as fold enrichment relative to an isotype IgG control antibody.

B PAX6 and *Paupar* co-occupy a specific set of genomic binding sites. ChIP assays were performed in N2A cells using either an antibody against PAX6 or an isotype-specific control. The indicated DNA fragments were amplified using qPCR. Fold enrichment was calculated as 2^−ΔΔ*Ct*^ (IP/IgG).

C, D *Paupar* binding sites act as transcriptional regulatory elements. N2A cells were transfected with the indicated reporter constructs in a luciferase assay. Luciferase activity was compared to that of the empty SV40 promoter construct.

E–G *Paupar* transcript modulates the transcriptional activity of its binding sites in *trans*. Luciferase reporters were co-transfected into N2A cells together with either a non-targeting control or two independent *Paupar* targeting shRNA expression vectors. *Paupar* depletion was confirmed using qRT-PCR. For these reporter assays, a *Renilla* expression vector was used as a transfection control and the total amount of DNA transfected in each case was made equal. Data information: Results are presented as mean values ± s.e., *n* = 3 (A–D) or *n* = 4 (E–G); ***P < 0.001, **P < 0.01, *P < 0.05, one-tailed *t*-test, unequal variance.

We next measured PAX6 occupancy at a set of *Paupar* binding sites within the regulatory regions of genes whose expression changes in *Paupar* knockdown cells (Fig6B). Using chromatin immunoprecipitation (ChIP), we found a strong enrichment of PAX6 at *Paupar* binding sites within the regulatory regions of genes whose expression changes significantly upon both *Pax6* and *Paupar* knockdown. By contrast, the majority of the assayed *Paupar* binding locations associated with genes that changed in expression upon *Paupar* but not *Pax6* knockdown showed little enrichment for PAX6 (Fig6B).

Several lncRNAs titrate DNA binding transcription factors away from their genomic targets (Hung *et al*, 2011; Rapicavoli *et al*, 2013; Sun *et al*, 2013). We therefore examined PAX6 occupancy at a number of *Paupar* binding sites following *Paupar* knockdown and discovered that *Paupar* depletion does not significantly affect PAX6 chromatin occupancy at the regions tested (Supplementary Fig S7). In a similar manner, the androgen receptor (AR)-associated lncRNAs *Prncr1* and *Pcgem1* do not affect AR DNA binding but instead function to recruit transcriptional co-factors to DNA bound AR (Yang *et al*, 2013). Taken together, these findings indicate that PAX6 and *Paupar* specifically co-occupy a subset of *Paupar* binding sites associated with genes whose expression change upon *Pax6* and *Paupar* knockdown, and that PAX6 protein plays a role in targeting *Paupar* to the genome.

### *Paupar* modulates in *trans* the activity of transcriptional regulatory elements of neuro-developmental genes

We next tested whether *Paupar* CHART-Seq peaks function as transcriptional regulatory elements. To do this, we first selected 11 *Paupar* binding sites, ranging from 170 bp to 2.5 kb in length, located within the regulatory regions of genes controlling neural growth and differentiation (*E2f2*, *E2f7* [2 peaks], *Cdc6*, *Cdkn2c*, *Kdm7a* [2 peaks], *Sox1*, *Sox2*, *Hoxa1*, *Hes1*) and cloned each upstream of a heterologous SV40 promoter in a pGL3 luciferase vector. All of these genes, except *Sox2* whose expression is undetectable in N2A cells, showed evidence of being regulated by *Paupar* using microarray profiling. The transcriptional activity of these constructs was then compared to that of the SV40 promoter alone following transient transfection in N2A cells. One (*E2f2*BS) out of six focal peaks of < 600 bp in length strongly activated (3.9-fold) the SV40 promoter, a further three displayed a small, but significant (1.3–1.7-fold), increase in SV40 promoter activity (Fig6C), while four out of five broad peaks > 1 kbp (Fig6D) reproducibly repressed the SV40 promoter (1.6–1.8-fold). Consistent with the observed enrichment of *Paupar* CHART-Seq peaks at neuronal DNase I HS sites and *cis*-regulatory elements (Fig5) these results indicate that *Paupar* binding sites function in transcriptional control and can operate as both transcriptional enhancers and repressors.

Finally, we examined whether the *Paupar* transcript plays a *trans*-acting role in modulating the transcriptional response of its genomic binding sites. For this we chose to investigate the dependence on the *Paupar* transcript of the transcriptional activity of the strong *E2f2* enhancer element as well as the weak *E2f7* activating region and the repressor elements associated with the *Sox2*, *Hes1* and *Hoxa1* genes. To achieve this, reporters were co-transfected into N2A cells along with either a non-targeting control or two independent *Paupar*-targeting shRNA expression vectors in a luciferase assay. Strikingly, depletion of the endogenous *Paupar* transcript led to a dose-dependent increase in the enhancer activity of the SV40-*E2f2*BS reporter (Fig6E and F). In accordance with this, *E2f2* displayed one of the greatest expression changes (1.9-fold up-regulation; Supplementary Table 2) in our transcriptome profiling of *Paupar* knockdown cells. The activity of the SV40-*E2f7*BS reporter was not altered by *Paupar* loss of function in this assay. Furthermore, *Paupar* depletion suppressed the ability of both the SV40-*Sox2*BS and SV40-*Hes1*BS reporters to function as transcriptional repressors, in a manner dependent on the levels of the *Paupar* transcript, while the repressive function of the SV40-*Hoxa1*BS reporter was not altered (Fig6G). These findings demonstrate a transcriptional regulatory function for the *Paupar* transcript at its genomic binding sites in *trans* and reveal that a lncRNA can function at transcriptional regulatory regions on different chromosomes distinct from its site of synthesis to control target gene expression.

## Discussion

The extent by which lncRNAs contribute to genome function remains unclear. Here, we have used shRNA mediated knockdown, microarray profiling, genome-wide mapping and reporter assays to detail the RNA-dependent mode of action of *Paupar*, a chromatin-associated CNS expressed lncRNA transcript, in the regulation of gene transcription. Our findings that the *Paupar* transcript acts both locally, to regulate *Pax6* expression, and distally in a transcript- and *Pax6*-dependent manner, reveal that lncRNAs can be functionally complex and will not always fall exclusively into *cis*- or *trans*-acting categories. Our data indicate that the *Paupar* transcriptional response is driven by at least three contributing components: downstream gene expression changes arising from *Paupar* regulation of *Pax6* expression; *Pax6*- and *Paupar*-dependent *trans-*acting gene expression changes likely mediated through a physical interaction between *Paupar* and PAX6 protein at *Paupar* associated loci; and *trans-*acting *Pax6*-independent *Paupar* functions at many bound transcriptional regulatory regions genome-wide.

Consistent with a role for *Paupar* in regulating *Pax6* expression, the *Paupar* locus has characteristics of a transcriptional enhancer. It spans both a previously defined *Pax6* neuroretina specific enhancer conserved between human and quail (Plaza *et al*, 1999) and a known N2A cell DNase I HS site (McBride *et al*, 2011). Furthermore, ENCODE data indicate its locus to be marked by a high ratio of histone H3K4me1 compared to H3K4me3, with high levels of H3K27me3 and an absence of H3K27ac in the mouse E14.5 brain. Different classes of enhancer-associated *cis*-acting ncRNAs have been described to date. Enhancer RNAs (eRNAs) are relatively short polymerase II-transcribed, predominantly non-polyadenylated, bidirectional transcripts, first identified at neuronal enhancers (Kim *et al*, 2010) while enhancer-like lncRNAs are strand-specific, polyadenylated transcripts (De Santa *et al*, 2010; Orom *et al*, 2010) which in these respects are more similar to *Paupar*. The chromatin signature of the *Paupar* locus, along with the finding that *Paupar* regulates *Pax6* expression, implies that *Paupar* may play an important role in nervous system development. Furthermore, the widespread effect on over 900 genes when *Paupar* transcript levels were reduced by 52% may be associated with the haploinsufficiency and dosage-sensitivity of *Pax6* (Georgala *et al*, 2011).

We show that *Paupar* associates with approximately 2800 sites in the genome and, where tested, these regions operate as transcriptional regulatory elements whose activity can be modulated by *Paupar* transcript levels. Our data are consistent with a model in which *Paupar* is indirectly targeted to the genome through RNA-protein interactions with multiple different neural transcription factors including PAX6. In accordance with this, we discovered a motif resembling a known PAX6 DNA binding motif within approximately 9% of the 500 top-scoring *Paupar* bound sequences and showed that *Paupar* and PAX6 co-occupy specific genomic sites within the regulatory regions of genes whose expression change upon both *Pax6* and *Paupar* knockdown. Furthermore, our data show that *Paupar* does not affect PAX6 chromatin occupancy and suggest that *Paupar* may regulate the association of PAX6 with its transcriptional cofactors to control target gene expression.

It is likely that other nuclear enriched lncRNAs operate in a similar manner to *Paupar*. The *CTBP1-AS* lncRNA has recently been demonstrated to possess *cis-* as well as *trans-*acting functions (Takayama *et al*, 2013) while *Prncr1* and *Pcgem1* have been shown to bind the AR and associate with androgen responsive enhancers genome-wide (Yang *et al*, 2013). *Hotair* and *Terc* occupy hundreds of short genomic regions of up to 1 kb in length across multiple chromosomes while Drosophila *roX2* interacts with Chromosome Entry Sites on the X-chromosome (Chu *et al*, 2011; Simon *et al*, 2011), regions that can recruit the dosage compensation machinery when inserted into autosomes (Fagegaltier & Baker, 2004). Human Alu RNA, transcribed from short interspersed elements, binds polymerase II and therefore has the potential to function as a general transcriptional repressor (Mariner *et al*, 2008) while the lateral mesoderm specific lncRNA *Fendrr* interacts *in vitro* with *Foxf1* promoter fragments in *trans* (Grote *et al*, 2013). We therefore propose that *Paupar* is a member of a class of nuclear enriched lncRNAs that can interact with multiple transcriptional enhancers and silencers to regulate gene expression in *trans* in a transcript-dependent manner.

LncRNAs that play important roles in the development and function of the nervous system may be dysregulated in neurological disorders. We have shown that *Paupar* loss of function disrupts the normal cell cycle profile of N2A neuroblastoma cells and induces neuronal differentiation. Furthermore, its transcript binds and modulates the activity of *Sox2*, *Hes1* and *E2f2* gene transcriptional regulatory elements in *trans*, in addition to regulating the expression of *Paupar*'s adjacent *Pax6* gene. Although the roles of *Sox2* and *Hes1* in neural progenitor cell maintenance and neurogenesis are well characterised (Pevny & Nicolis, 2010; Kageyama *et al,* 2008), the function of the *E2f2* gene in neural lineages has been less well studied. We found that *E2f2*, and the related *E2f7* and *E2f8* genes, are up-regulated upon *Paupar* knockdown in our profiling experiments and that the *Paupar* transcript operates to restrict the activity of an *E2f2* enhancer element. *E2f2* functions as a pro-survival factor during retinal development (Chen *et al*, 2009) and is involved in maintaining the differentiated state of post-mitotic neurons in cells in culture (Persengiev *et al*, 2001). Furthermore, *E2f2* over-expression promotes cell cycle arrest and inhibits S-phase progression in PC12 neurons (Persengiev *et al*, 2001). Up-regulation of *E2f* family members in *Paupar* knockdown N2A cells may thus contribute to the accumulation of cells in S and G2 phases of the cell cycle and the observed neural differentiation phenotype.

The mechanisms of action of a growing number of *cis*-acting lncRNAs have been studied and have revealed roles for both the RNA molecule and lncRNA transcription in regulating the expression of genomically neighbouring genes (Lai *et al*, 2013; Melo *et al*, 2013; Wang *et al*, 2011). By way of contrast, we have uncovered the mode of action of a lncRNA molecule that is able to regulate the expression of genomically local as well as distal genes and suggest that lncRNAs which modulate cellular functions via genome-wide targets may be more widespread than previously anticipated.

## Materials and Methods

### Plasmid construction

To generate shRNA expression plasmids we first used the Whitehead Institute siRNA selection program to design shRNAs targeting multiple regions of *Paupar* and *Pax6*. Selected sequences were filtered to eliminate off-target effects by performing a BLAST search of the NCBI RefSeq database and removing hits with >15 matched bases of the anti-sense strand. Double stranded DNA oligonucleotides containing sense-loop-antisense targeting sequences were then cloned downstream of the U6 promoter in pBS-U6-CMVeGFP (Sarker *et al*, 2005) by linker ligation. To generate the *Paupar* expression plasmid the full length *Paupar* transcript was PCR amplified as a *Xho*I fragment from mouse N2A cell cDNA and inserted into pCAGGS. *Paupar* CHART-Seq peak regions were PCR cloned from N2A genomic DNA and inserted upstream of the SV40 promoter in pGL3-Pro (Promega) to generate a panel of luciferase reporters to test for transcriptional regulatory activity. The sequences of all oligonucleotides used for cloning are shown in Supplementary Table 1.

### Cell culture

N2A mouse neuroblastoma cells were grown in DMEM supplemented with 10% fetal bovine serum. E14 mouse ES cells were grown on 0.1% gelatin-coated dishes in DMEM supplemented with 15% fetal calf serum, Leukemia-Inhibitor factor, 1× non-essential amino acids, 2 mM l-glutamine, 50 mg/ml penicillin/streptomycin and 50 μM 2-mercaptoethanol. To induce neuronal differentiation ES cells were seeded onto gelatinised plates and grown in differentiation medium (DMEM supplemented with 10% fetal calf serum, 1× non-essential amino acids, 2 mM l-glutamine, 50 mg/ml penicillin/streptomycin, 50 μM 2-mercaptoethanol and 10^−7^ M all*-trans* retinoic acid).

### Transcriptomic analysis

Total RNA was isolated using the Qiagen Mini RNeasy kit following the manufacturer's instructions and RNA integrity assessed on a BioAnalyzer (Agilent Technologies). 200 ng RNA was used to generate labelled sense single stranded DNA (ssDNA) for hybridization with the Ambion WT Expression Kit, the Affymetrix WT Terminal Labelling and Controls Kit and the Affymetrix Hybridization, Wash, and Stain Kit as described by the manufacturer. Sense ssDNA was fragmented and the distribution of fragment lengths was measured on a BioAnalyzer. Fragmented ssDNA was then labelled and hybridized to the Affymetrix GeneChip Mouse Gene 1.0 ST Array (Affymetrix). Chips were processed on an Affymetrix GeneChip Fluidics Station 450 and Scanner 3000. Affymetrix CEL files were analysed using the Limma, oligo, and genefilter R Bioconductor packages (Carvalho & Irizarry, 2010; Smyth, 2004). Arrays were RMA background corrected, quantile normalised and summary expression values calculated for Refseq and full length mRNAs. Genes changing upon *Paupar* knockdown were filtered to remove genes showing little variation in expression (variance cut off of 0.5), while for the *Pax6* knockdown analysis, genes with consistently low expression were removed before the identification of significant changes. In each case, differential expression between three knockdown and three control samples (biological replicates) was tested using the Limma Ebayes algorithm. Gene Ontology analyses were performed using GOToolBox, and representative significantly enriched categories selected from a hypergeometric test with a Benjamini-Hochberg corrected *P*-value threshold of 0.05 (http://genome.crg.es/GOToolBox/).

### CHART-seq and analysis

CHART Enrichment and RNase H Mapping experiments were performed as previously described (Simon, 2013). CHART extract was prepared from approximately 8 × 10^7^ N2A cells per pull down and hybridized with 810 pmol biotinylated oligonucleotide cocktail (Supplementary Table 1) overnight with rotation at room temperature. Complexes were captured using 250 μl MyOneC1 streptavidin beads (Invitrogen) overnight at room temperature with rotation. Bound material was extensively washed and eluted using RNase H (New England Biolabs) for 30 min at room temperature. Samples were treated with Proteinase K and cross-links were reversed. RNA was purified from 1/5 total sample volume using the Qiagen miRNeasy kit while DNA was purified from the remaining sample by Phenol:CHCl_3_:isoamyl extraction and ethanol precipitation. DNA was sheared to an average fragment size of 150–300 bp using a Bioruptor (Diagenode) and sequenced on an Illumina HiSeq.

CHART-seq was performed in replicate with two independent pull down samples and matched controls using non-targeting LacZ oligos. A single sample of input DNA from N2A cells was prepared and sequenced separately. 50 bp, paired-end reads were mapped to the mouse genome (mm9) using bowtie with the options ‘–m1 –v2 –best –strata –a’. For each *Paupar* sample, peaks were called against the matched LacZ control and against the N2A input sample. Peak calls were made using the MACS2 algorithm (Zhang *et al*, 2008; https://github.com/taoliu/MACS/blob/master/README) with the options ‘–mfold 10 30 –gsize=2.39e9 –qvalue=0.01’ using the CGAT pipeline ‘pipeline_mapping.py’ (https://github.com/CGATOxford/cgat). Peak calls were then filtered such that only peak calls with a −log10 *q* value > 5 were retained (FDR 0.001%).

### Characterisation of *Paupar* binding sites

The chromosomal distribution of *Paupar* peaks was visualised using the R Bioconductor package ‘ggbio’ (Yin *et al*, 2012). Genome territory enrichments were identified using the Genome Association Tester (GAT; Heger *et al*, 2013), using a mappability filtered workspace, an isochore file partitioning the genome into 8 bins based on regional GC content and 10,000 simulations. Chromosomal enrichments were analysed by proportionally assigning chromosomal territories to a single virtual meta-chromosome before using GAT to test for GC and mappability corrected enrichments as before. Peak shapes were visualised using read count normalised (MACS2–SPMR), background subtracted (MACS2–bdgcmp) coverage tracks from which regions covering peaks were extracted and centred based on the location of the peak maximum. Gene ontology categories enriched for *Paupar* binding were identified by intersecting regulatory regions for known coding genes with *Paupar* binding sites. Regulatory regions for genes were defined as a basal domain surrounding the TSS of −5 kb to +1 kb plus an extended domain of upstream and downstream to the nearest gene's basal domain or to a maximum distance of 1 Mb, following the GREAT definition (McLean *et al*, 2010). Enrichments were identified with GAT using the regulatory regions of all genes as the workspace, and 10,000 simulations. Because we noted some correspondence between *Paupar* binding and gene expression level, we supplied GAT with a file stratifying the workspace into six bins based on gene expression level in N2A cells under the ‘–isochore’ option to conservatively avoid associations solely due to expression level.

*Paupar* peaks were characterised using DNase I hypersensitivity sites identified by the Stamatoyannopoulos lab at the University of Washington and regulatory elements identified by the Ren lab at the Ludwig Institute for Cancer Research (ENCODE Project Consortium, 2011). Enrichments of DNase I HS and regulatory elements overlapping *Paupar* peaks were assessed using GAT to control for mappability and regional GC content as before.

Complementarity of *Paupar* sequence and binding locations was assessed using the EMBOSS Water algorithm (Rice *et al*, 2000) to perform Smith-Waterman alignment with a range of gap opening and extension penalties. De novo motif discovery was performed using the MEME-ChIP (Machanick & Bailey, 2011) algorithm to examine the unmasked DNA sequence of the central regions of top scoring (MACS2 peak score) peak locations. MEME-ChIP was run with the options ‘-meme-mod zoops -meme-minw 5 -meme-maxw 30—meme-nmotifs 50’ using a custom background file prepared from regions flanking the peak locations using the command ‘fasta-get-markov -m 2’. Enrichment of known vertebrate transcription factor binding sites from the TRANSFAC Professional database (Matys *et al*, 2006) was assessed using the AME algorithm (McLeay & Bailey, 2010) with the options ‘–method fisher –length-correct’ using the sequence and background file prepared for MEME-ChIP analysis.

### *Paupar* knockdown and flow cytometry

Approximately 2 × 10^5^ cells were plated per well in a six well plate. 16–24 h later cells were transfected with 1.5 μg shRNA expression construct using FuGENE 6 (Promega) according to the manufacturer's instructions. Total RNA was extracted from the cells 2–3 days later using the Qiagen Mini RNeasy kit according to the manufacturer's instructions. For stable transfections, N2A cells were co-transfected with a 5:1 ratio of pBSU6-sh408 expression vector and pTK-Hyg (Clontech). Three days after transfection 200 μg/ml Hygromycin B was added to the cells and individual drug resistant clones were isolated and expanded under selection conditions. Individual clones were characterized for *Paupar* expression using qRT-PCR. For flow cytometry, cells were harvested by trypsinization, washed twice with PBS and fixed as a single cell suspension in −20°C filtered 70% ethanol. After incubation at 4°C for 10 min cells were pelleted, treated with 40 μg/ml RNase A and propidium iodide (40 μg/ml) for 30 min at room temperature and then analysed using a FACSCalibur (BD Biosciences) flow cytometer.

### qRT-PCR and RACE

The QuantiTect Reverse Transcription Kit (Qiagen) was used for reverse transcription and followed by SYBR Green quantitative PCR using a Step One Plus Real-Time PCR System (Applied Biosystems). RACE was performed using the GeneRacer Kit (Invitrogen) following the manufacturer's instructions. Human foetal brain RNA was obtained from Promega. Primers are shown in Supplementary Table 1.

### Cell fractionation

Approximately 2.5 × 10^6^ cells were pelleted, washed with PBS, resuspended in 250 μl Lysis Buffer (15 mM HEPES pH7.5, 10 mM KCl, 5 mM MgCl_2_, 0.1 mM EDTA, 0.5 mM EGTA, 250 mM Sucrose, 0.4% Igepal, 1 mM DTT, 40 U/ml RNaseOUT (Invitrogen), protease inhibitor cocktail [Roche]) and incubated on ice for 20 min. Nuclei were centrifuged at 2,000 *g* for 10 min at 4°C and the supernatant was collected as the cytoplasmic fraction. Nuclei were then resuspended in 50 μl Nuclei Lysis Buffer (10 mM HEPES pH7.5, 0.1 mM EDTA, 0.1 mM EGTA, 1 mM DTT, 40 U/ml RNaseOUT (Invitrogen), protease inhibitor cocktail [Roche]) and incubated on ice for 5 min. Nuclei were pelleted at 17,000 *g* for 5 min at 4°C and the supernatant was removed as the nucleoplasm fraction. The pellet was then resuspended in 50 μl Salt Extraction Buffer (25 mM HEPES pH7.5, 10% glycerol, 420 mM NaCL, 5 mM MgCl_2_, 0.1 mM EDTA, 1 mM DTT, 40 U/ml RNaseOUT (Invitrogen), protease inhibitor cocktail [Roche]) and incubated for 30 min at 4°C with rotation. The sample was then centrifuged at 17,000 *g* for 20 min at 4°C. The supernatant was collected as the salt extracted fraction and the pellet resuspended in 50 μl Salt Extraction Buffer to generate the chromatin fraction. RNA was isolated from each fraction using the Qiagen Mini RNeasy kit following the manufacturer's instructions.

### UV-RIP

UV-RIP was performed as described in (Zhao *et al*, 2010) with minor modifications. Approximately 1 × 10^7^ N2A cells per UV-RIP were UV crosslinked in ice-cold PBS at 254 nm, 120 mjoules/cm^2^ using a Stratalinker (Stratagene). Nuclei were isolated, disrupted by sonication (three cycles, 30 sec ON/OFF) using a Bioruptor (Diagenode) and treated with 20 μl Turbo DNase (Ambion) before overnight incubation with either anti-rabbit PAX6 (AB2237) or rabbit IgG (both Millipore) polyclonal antibodies. Complexes were captured using Protein-A magnetic beads (Pierce), washed using low- and high-stringency buffers and then treated with RNA grade Proteinase K (Invitrogen). RNA was extracted using Trizol (Invitrogen) and analysed by qRT-PCR.

### ChIP

ChIP was performed using approximately 1 × 10^7^ N2A cells per assay. Cells were trypsinized, resuspended in 10 ml PBS containing 1% final concentration formaldehyde and incubated for 10 min at room temperature with rotation. Cross-linking reactions were quenched with 0.125 M glycine for 5 min at room temperature and washed twice with ice-cold PBS. Nuclei were then isolated and chromatin was sheared to approximately 500 bp using a Bioruptor (Diagenode). Cross-linked chromatin was immunoprecipitated using 5 μg anti-rabbit PAX6 or anti-rabbit IgG control antibodies (both Millipore) overnight at 4°C. Complexes were collected using Protein-A magnetic beads (Pierce) pre-blocked with BSA (New England Biolabs) and transfer RNA (Roche), then washed and eluted. Cross-links were reversed at 65°C overnight and DNA was precipitated, treated with Proteinase K (Roche) and then purified using a PCR Purification Kit (Qiagen).

### Luciferase assays

Approximately 5 × 10^4^ N2A cells were seeded per well in a 12-well plate. On the next day, cells were transfected with the indicated ratios of reporter constructs and expression vectors using FuGENE 6 (Promega) according to the manufacturer's instructions. The pRL-tk plasmid (Promega) was co-transfected into each well to normalize for transfection efficiency. The total amount of DNA was made up to 1 μg for each transfection by the addition of empty expression vector. Forty-eight hours after transfection lysates were prepared and assayed for firefly and renilla luciferase activity.

### Data deposition

Microarray and CHART-Seq data have been deposited in the GEO database under accession number GSE52571 (http://www.ncbi.nlm.nih.gov/geo/query/acc.cgi?acc=GSE52571).

## Supporting Information

Supplementary Table S7

Review Process File
