# Supplementary material for: The long non-coding RNA Paupar regulates the expression of both local and distal genes
Source: EMBO J. 2014 Feb 1;33(4):296–311. doi: 10.1002/embj.201386225 (PMC3983687; doi:10.1002/embj.201386225)
Supplement: Supplementary file 1 [file embj0033-0296-sd1.pdf]

Vance Supplemental Fig S1

A

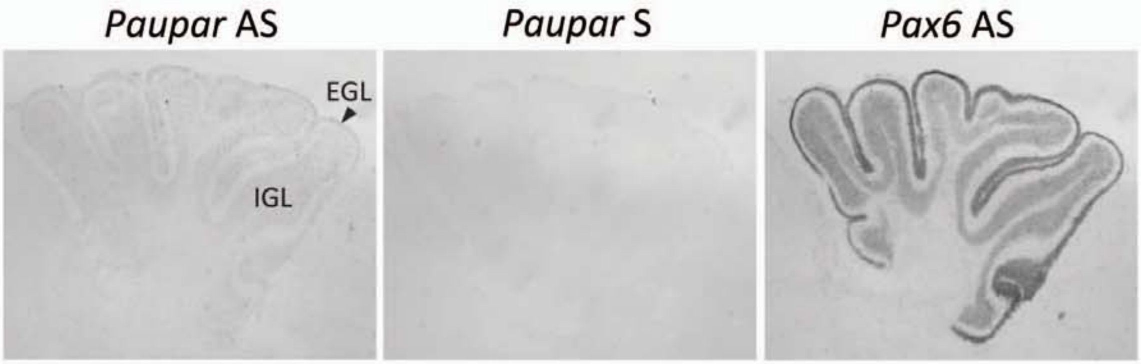

B

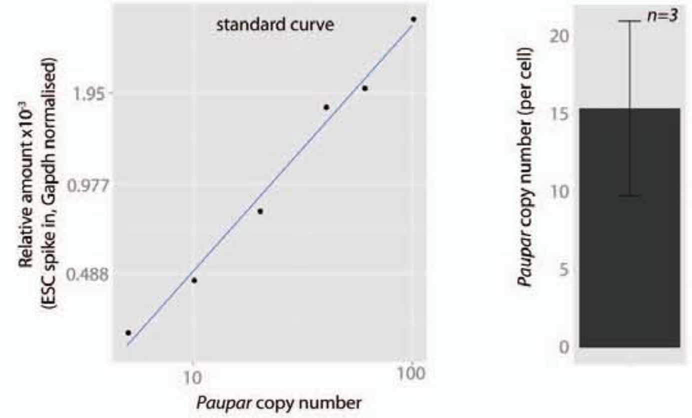

C

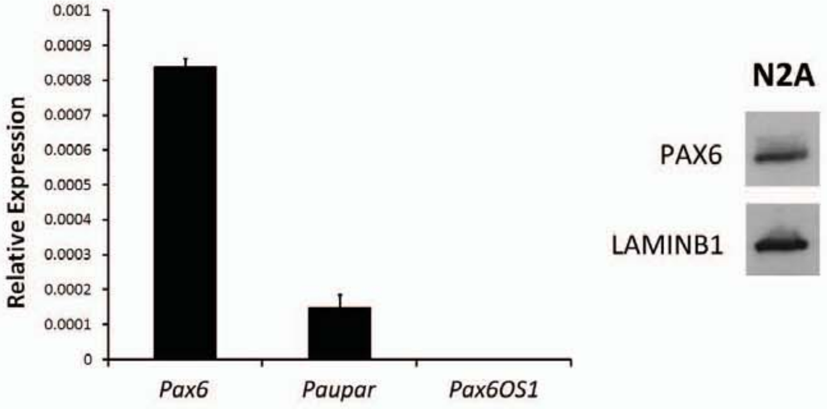

Figure S1 Characterisation of *Paupar* expression. (A) *Paupar* and *Pax6* in situ hybridisation in the mouse cerebellum. An antisense (AS) *Paupar* riboprobe reveals expression of the transcript in the internal granular and external germinal layers (IGL / EGL) of the cerebellum at P7. The *Paupar* (AK032637) transcript was first sequenced from neonate cerebellum. *Pax6* shows a similar pattern of expression although at a much higher level. The negative control sense (S) probe on an adjacent section is also shown. (*B*) *Paupar* is expressed at an estimated level of 15.4 ± 5.62 copies per cell in N2A cells. We generated a standard curve of known *Paupar* copy number by spiking RNA from ES cells, which do not express *Paupar*, with in vitro transcribed *Paupar* transcript (left). Mean *Paupar* expression per cell was calculated from three independent qRT-PCR experiments using RNA extracted from a defined number of cells. This value was used to estimate *Paupar* copy number from the standard curve. Mean copy number per cell +/- s.e. displayed (right).(C) Relative expression of *Pax6*, *Paupar* and *Pax6OS1* in N2A mouse neuroblastoma cells. Transcript levels were measured using qRT-PCR. Results are presented relative to *Gapdh* (left). PAX6 protein expression was detected in N2A cells by western blotting. A LAMINB1 antibody was used as a loading control (right).
