# Supplementary figures and images for: The long non-coding RNA Paupar regulates the expression of both local and distal genes
Source: EMBO J. 2014 Feb 1;33(4):296–311. doi: 10.1002/embj.201386225 (PMC3983687; doi:10.1002/embj.201386225)

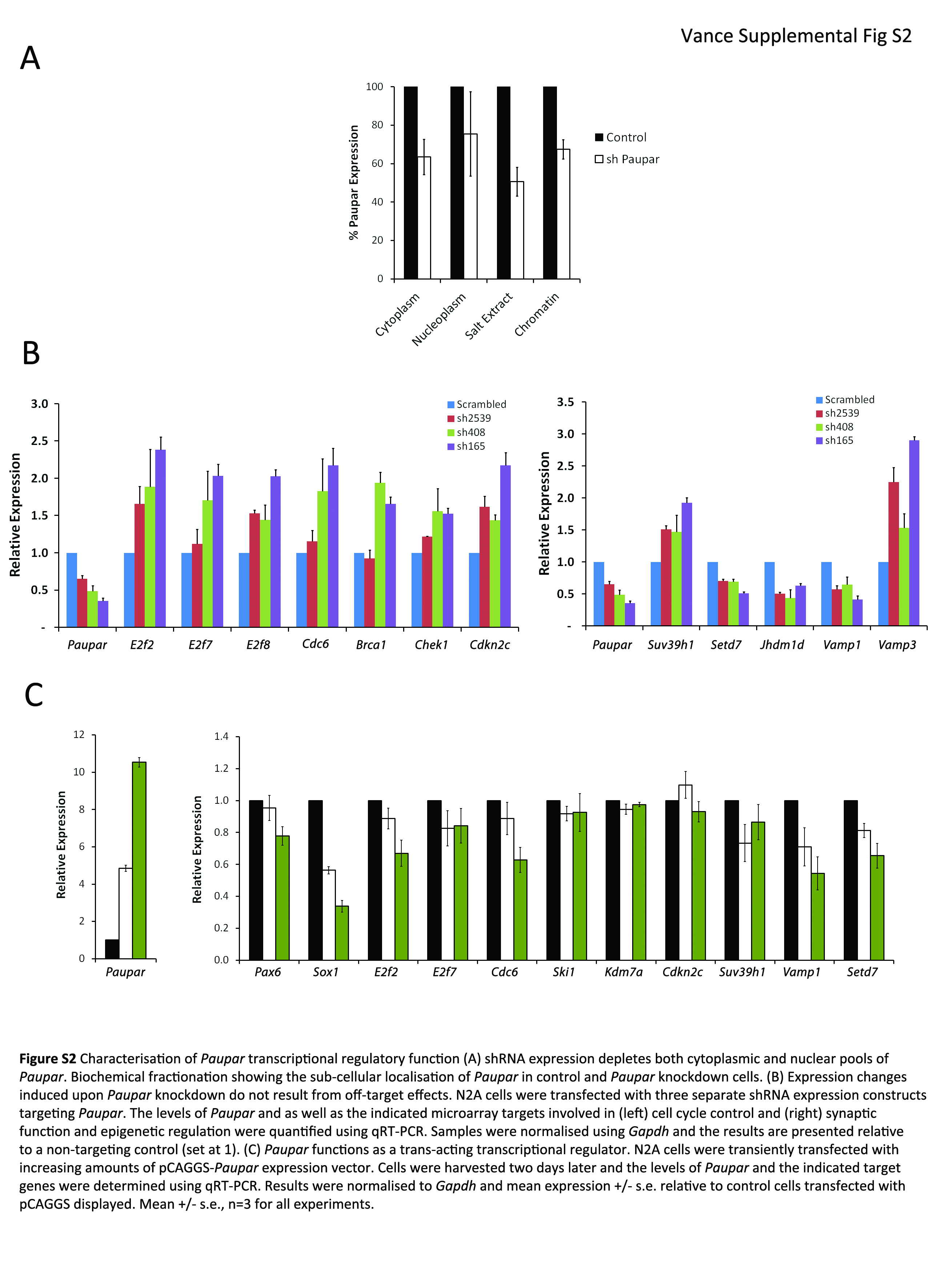

Supplement: Supplementary file 2 [file embj0033-0296-sd2.tif]

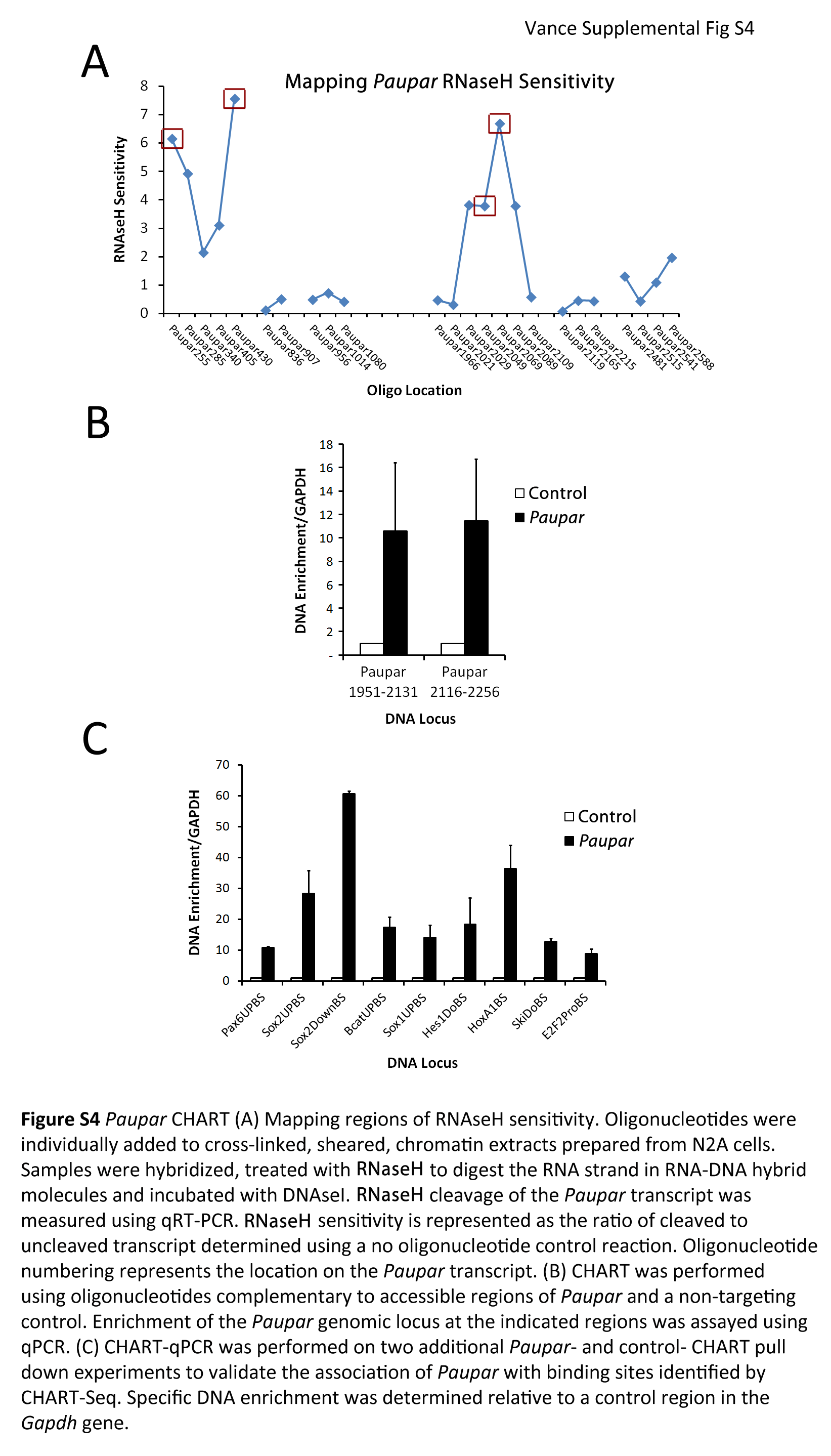

Supplement: Supplementary file 4 [file embj0033-0296-sd4.tif]

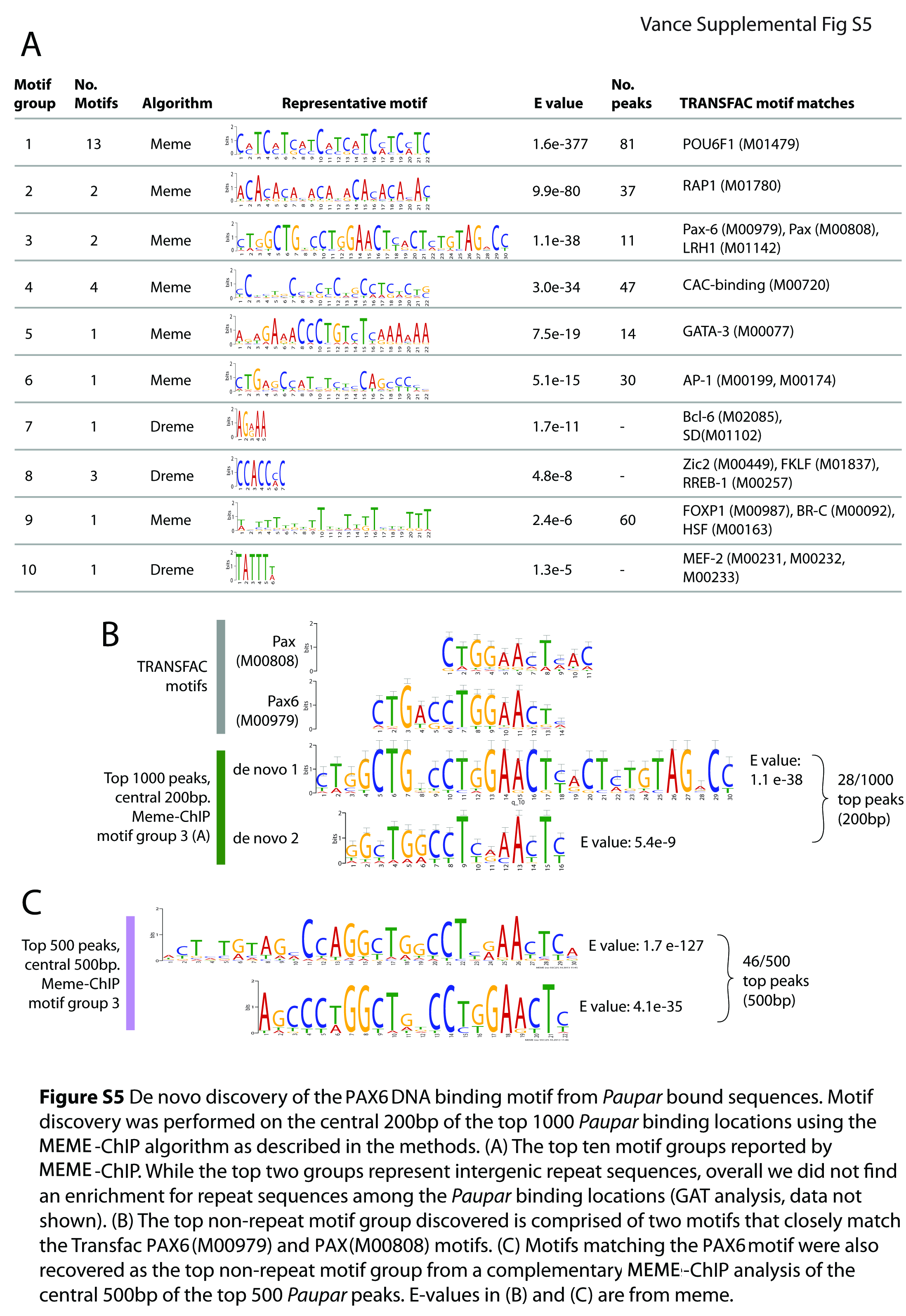

Supplement: Supplementary file 5 [file embj0033-0296-sd5.tif]

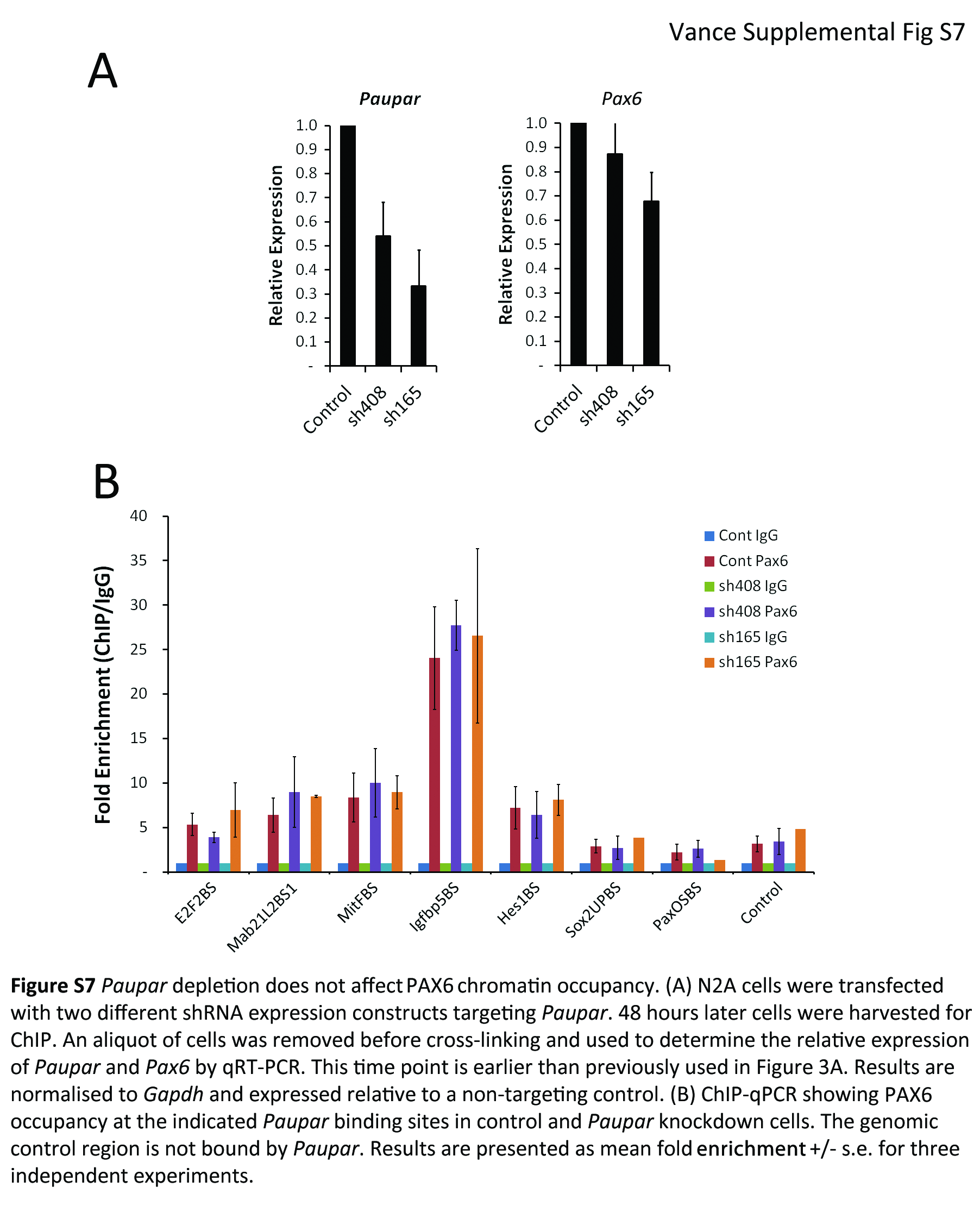

Supplement: Supplementary file 7 [file embj0033-0296-sd7.tif]
