# Supplementary material for: The long non-coding RNA Paupar regulates the expression of both local and distal genes
Source: EMBO J. 2014 Feb 1;33(4):296–311. doi: 10.1002/embj.201386225 (PMC3983687; doi:10.1002/embj.201386225)
Supplement: Supplementary file 3 [file embj0033-0296-sd3.pdf]

Vance Supplemental Fig S3

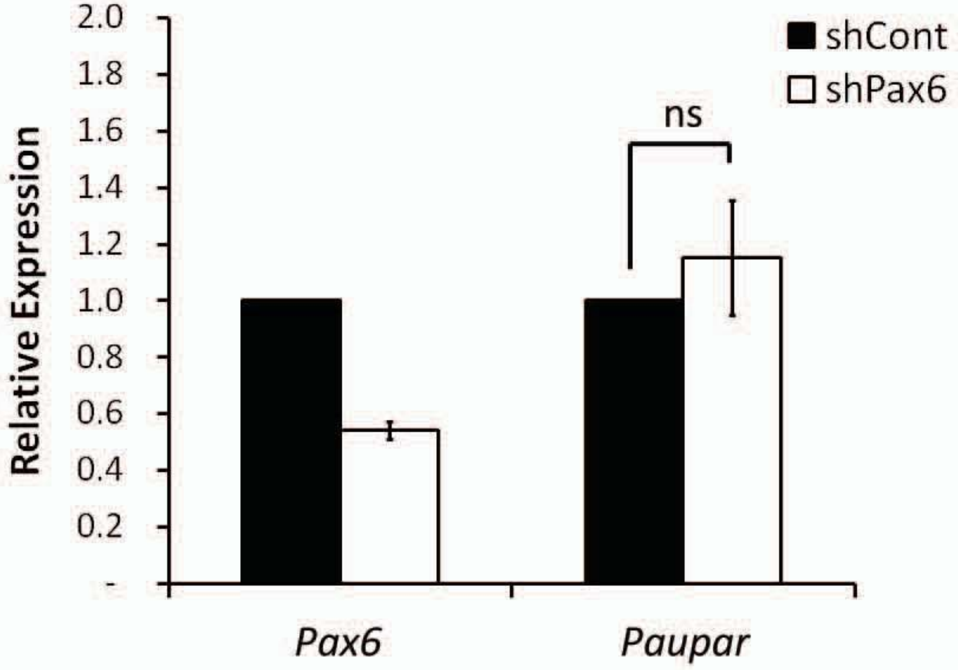

Figure S3 *Paupar* levels do not change significantly upon *Pax6* knockdown. N2A cells were transfected with either a shRNA expression construct targeting *Pax6* or a non-targeting control. *Pax6* and *Paupar* transcript levels were quantified using qRT-PCR three days later. Samples were normalised using *Gapdh* and results are presented relative to the non-targeting control (set at 1). Bars represent mean values +/- s.e., n=3.
