# Supplementary material for: The long non-coding RNA Paupar regulates the expression of both local and distal genes
Source: EMBO J. 2014 Feb 1;33(4):296–311. doi: 10.1002/embj.201386225 (PMC3983687; doi:10.1002/embj.201386225)
Supplement: Supplementary file 6 [file embj0033-0296-sd6.pdf]

Vance Supplemental Fig S6

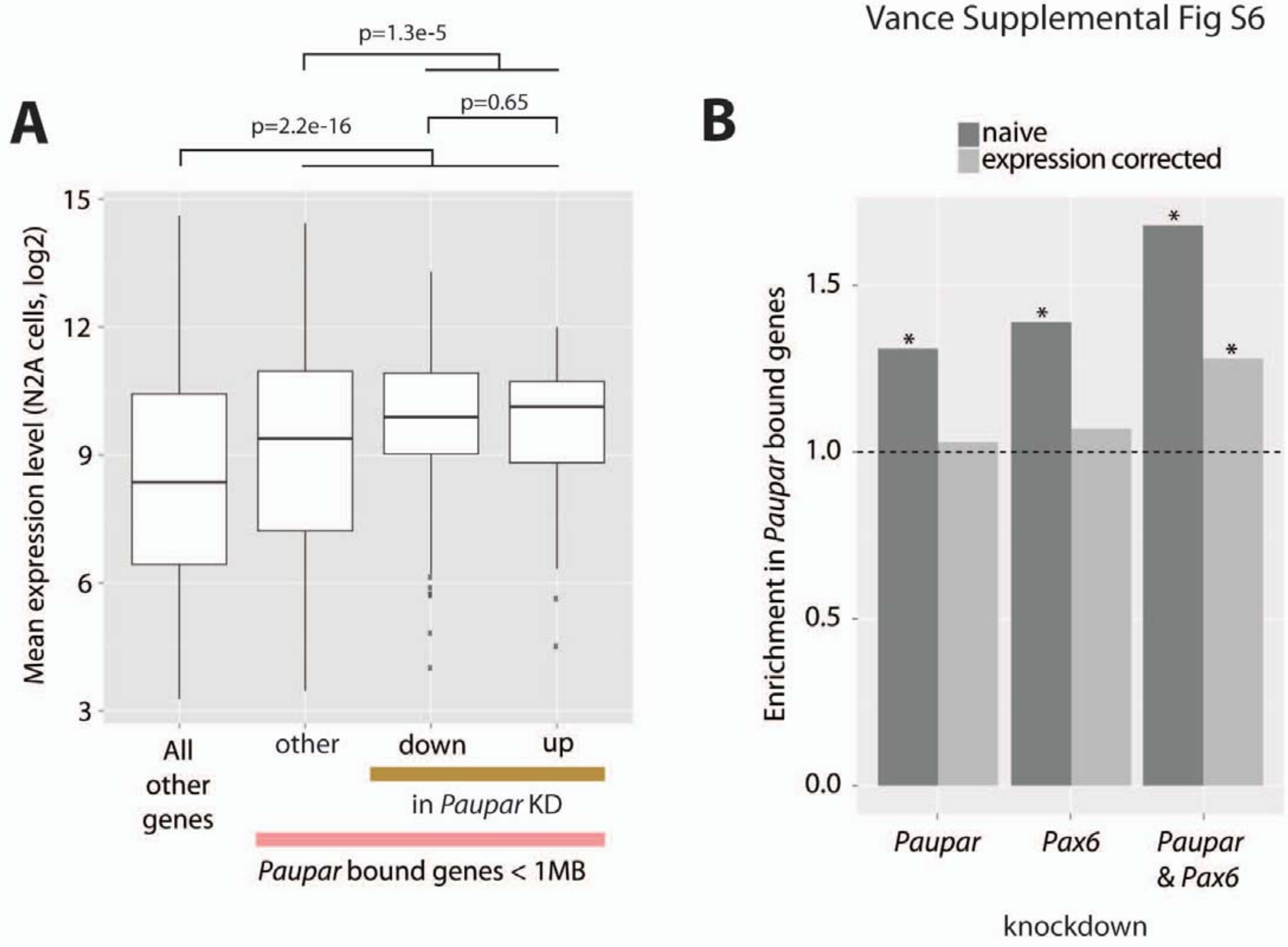

Figure S6 Expression levels and enrichments of *Paupar* associated genes for *Paupar* and/or *Pax6* regulated genes. *Paupar* bound genes were identified using the GREAT definition of gene promoters as described in the methods. (A) Genes bound and regulated by *Paupar* are more highly expressed in N2A cells based on the control microarray data from the *Paupar* and *Pax6* knock-down experiments (p-values for the indicated comparisions were calculated using a two-sided Mann-Whitney U test). (B) The enrichment of *Paupar* and/or *Pax6* regulated genes among genes associated with *Paupar* binding was tested using the Genomic Association Tester (GAT). Both naive (only mapability corrected) and gene expression level corrected scores reveal a significant enrichment of *Paupar* and *Pax6* regulated genes among those associated with *Paupar* binding. For the expression level correction, the analysis was stratified by gene expression level using six equal bins of expression level. Asterisks indicate a significant enrichment (BH corrected p value < 0.05).
