# Supplementary material for: The long non-coding RNA Paupar regulates the expression of both local and distal genes
Source: EMBO J. 2014 Feb 1;33(4):296–311. doi: 10.1002/embj.201386225 (PMC3983687; doi:10.1002/embj.201386225)
Supplement: Supplementary file 15 [file embj0033-0296-sd15.pdf]

THE
EMBO
JOURNAL

Manuscript EMBO-2013-86225

The long non-coding RNA Paupar regulates the expression of both local and distal genes

Keith W Vance, Stephen N Sansom, Sheena Lee, Vladislava Chalei, Lesheng Kong, Sarah E Cooper, Peter L Oliver and Chris Ponting

Corresponding author: Keith Vance & Chris Ponting, University of Oxford

Review timeline:

Submission date:	17 July 2013
Editorial Decision:	08 August 2013
Revision received:	28 October 2013
Editorial Decision:	15 November 2013
Revision received:	21 November 2013
Accepted:	22 November 2013

Editor: Anke Sparmann & Anne Nielsen

Transaction Report:

(Note: With the exception of the correction of typographical or spelling errors that could be a source of ambiguity, letters and reports are not edited. The original formatting of letters and referee reports may not be reflected in this compilation.)

1st Editorial Decision 08 August 2013

Thank you for submitting your research manuscript entitled "The long non-coding RNA Paupar regulates the expression of both local and distal genes" (EMBOJ-2013-86225) to our editorial office. It has now been seen by three referees and their comments are provided below.

All reviewers appreciate your study and are in general supportive of publication in The EMBO Journal. Nevertheless, they do raise a number of important concerns, and emphasize that a significant revision of the manuscript will be required. Although several issues can be addressed by textual changes, this will also entail additional - in certain cases challenging - experimentation.

Given the comments provided, I would like to invite you to submit a suitably revised manuscript to The EMBO Journal that attends to the raised concerns in full. I should add that it is our policy to allow only a single major round of revision and that it is therefore important to address the raised concerns at this stage. Please do not hesitate to contact me should any particular argument require further clarification.

When preparing your letter of response to the referees' comments, please bear in mind that this will form part of the Review Process File, and will therefore be available online to the community. For more details on our Transparent Editorial Process, please visit our website: http://www.nature.com/emboj/about/process.html

Thank you for the opportunity to consider your work for publication. As I will be leaving the journal shortly, my colleague Anne Nielsen will continue to handle your manuscript - she will have all

relevant information on file. We look forward to your revision!

REFEREE REPORTS

Referee #1

Vance et al report the characterization of Paupar, a lncRNA upstream of the Pax6 transcription factor genes. The authors show that Paupar is a chromatin-associated lncRNA enriched in brain and neural stem cells. Paupar appears to regulate the balance between proliferation and neuronal differentiation. Knockdown of Paupar and Pax6 suggest that Paupar affects Pax6 expression, and the two factors co-regulate target genes together. The authors further map the genome-wide binding sites of Paupar, and show that some of these elements are sufficient to confer repression in a transcript-dependent manner. Overall this is a significant contribution that should be published upon addressing the following points.

1. The authors make the claim that this work is the first to study distantly located lncRNA binding sites (p.3, and again in Discussion). However, this statement is incorrect with respect to well studied lncRNAs such as roX2, HOTAIR, and more recently Jpx. The first applicaton of genome-wide RNA location analysis was by Chu et al. and Simon et al. (cited by the authors). They mapped roX2 binding genome-wide, and found that roX2 occupied Chromosome Entry Sites (CES) on X-chromosome; CES are known to recruit the dosage compensation machinery in trans when knocked into autosomes. Jpx site of action was recently found to be a specific CTCF site. The text should be revised to give due credit tor prior work and avoid hyperboles (this paper is perfectly fine without them).

2. The authors were able to see effects with 52% reduction of Paupar level, which is a bit of a surprise. Is this related to the fact that Pax6 is haploinsufficient? The authors should at least comment on this unexpected sensitivity to Paupar level.

3. The broad Paupar binding sites can be up to 1 kb, which will contain many elements with complex and secondary effects. This should be discussed and at least acknowledged.

4. The de-repression of reporter genes upon Paupar knockdown and the fact that Paupar binds to Pax6 suggests that Paupar may titrate Pax6 or another positive factor away from DNA. This would similar to the mechanism described for PANDA and it partner NFYA, Jpx for CTCF, and Lethe for NF-kB. If the authors can test this idea it may unify several observations in this paper.

Referee #2

Vance et al. examine the functional potential of the conserved lncRNA Paupar, expressed upstream of, and antisense to, the key neuro-developmental regulator gene Pax6. Employing shRNA-mediated 'knockdown' of Paupar coupled to microarray profiling, the authors suggest its involvement in gene expression in neural cells, either by up- or down-regulation of significantly enriched genes categorized by their ontology. The morphological phenotype of Paupar knockdown cells agrees with Paupar playing a role in controlling neural developmental programs. In addition, it appears that Paupar represses the expression of the associated Pax6 gene in a transcript level-dependent manner. Since Pax6 is a major neuro-developmental regulator, the possibility is considered that part of the Paupar transcriptional response may be indirectly via Pax6, thus, common and distinct transcriptional targets are dissected. Interestingly, Paupar also has some Pax6-independent transcriptional targets. Importantly, the recently established CHART-Seq technology is used to map binding sites of Paupar RNA genome wide, addressing the question whether also a long-distance trans-regulator function is present. Indeed, ~2850 genome-wide binding sites are identified preferentially within promoters and 5'UTRs.

Major concerns

1. In order to demonstrate a role of Paupar as a trans-regulator, the authors utilize luciferase reporter assays and show that a few cloned candidate binding sites of Paupar provide regulatory potential,

which - in some cases - is also dependent on cellular levels of Paupar RNA. While implying a function of the respective Paupar binding sites, this analysis does not prove that the observed reporter regulation is exerted via direct binding of Paupar to the candidate regions. Since the stated novelty of this study is to verify a trans-acting role for a lncRNA, I think that more analysis is required at this point; e.g.
i) bioinformatics analysis of the Paupar binding sites aiming to identify any motif(s) that would support a significant 'recognition' by Paupar RNA (as also predicted by the demonstrated RNAseH sensitivity).
ii) mutagenesis of presented cases that would abrogate Paupar binding to DNA and consequently affect reporter regulation.
iii) in vitro validation of Paupar binding to identified DNA sequences (e.g. mobility shift assays).

2. One always wonders how ncRNAs that are supposed to associate with chromatin to exert their function can be downregulated by shRNAs. Would it not be expected that primarily the nonfunctional cytoplasmic (and perhaps nucleoplasmic) pool(s) would be depleted? At a minimum the author should demonstrate that levels of chromatin-associated Paupar declines upon shRNA administration.

3. What is the estimated copy number of Paupar per cell and is this compatible with the proposed function?

Referee #3

The manuscript by Vance et al. describes the characterization of a long noncoding RNA termed Paupar, which is predominantly expressed in the brain. The authors contend that Paupar could regulate both its neighboring genes particularly pax6 and additional genomic sites through a trans-mediated mechanism.

Major comments:

1-If the authors contend that Paupar functions in trans as is suggested by the CHART-seq data; then they should show that

A) Paupar over-expression effects the expression of similar gene-expression program as the KD of pauper. Off course the down regulated genes should be upregulated and vice versa.
B) Paupar over-expression (perhaps they could remove the 20 or so nucleotide targeted by shRNA) should rescue the cell cycle defect (Figure 2D) and neurite outgrowth Figure 2F.
C) They should perform structure/function analysis on Pauper determining the functional domains using expression analysis on Pax6 and other targets and whether the evolutionary conserved pieces are required for function.

2- the overlap between the pauper KD gene expression changes by array and the bound sites using pauper CAHRT is quite poor. From 3170 sites about 242 are effected by Paupar KD. Is there a difference between the sites that are functionally affected and those that not. Are the sites affected have a higher level of gene expression; Are they up or down regulated upon Paupar knock-down. It is difficult to know whether Paupar is an activator or repressor of transcription.

3- The authors show that Pauper interacts with Pax6, does it effect Pax6 chromatin residence? They could assess this by KD and over-expression of Paupar at many of Pax6 tagtes.

4- Frankly, it is difficult to interpret figure 6. Is there anything different between E2F2 site and E2F7 that result in differences in expression; Are there complementary sequences to Paupar in either of the putative Paupar binding sites. While the authors show that the E2F2BS site act as an activator (Figure 6A), Paupar KD result in increased expression Figure 2B, suggesting that Paupar is supposedly keeping the enhancer silent, this is a new mechanism of enhancer regulation which needs much more experiments to correctly decipher. Moreover, KD of Paupar resulting in the changes in SOX2BS and Hes1BS are so very small. I suggest removing this figure and performing complementary experiments with endogenous binding sites assessing gene expression changes and

changes in chromatin signatures.

1st Revision - authors' response 28 October 2013

Referee #1

Vance et al report the characterization of Paupar, a lncRNA upstream of the Pax6 transcription factor genes. The authors show that Paupar is a chromatin-associated lncRNA enriched in brain and neural stem cells. Paupar appears to regulate the balance between proliferation and neuronal differentiation. Knockdown of Paupar and Pax6 suggest that Paupar affects Pax6 expression, and the two factors co-regulate target genes together. The authors further map the genome-wide binding sites of Paupar, and show that some of these elements are sufficient to confer repression in a transcript-dependent manner. Overall this is a significant contribution that should be published upon addressing the following points.

Thank you for these positive comments.

1. The authors make the claim that this work is the first to study distantly located lncRNA binding sites (p.3, and again in Discussion). However, this statement is incorrect with respect to well studied lncRNAs such as roX2, HOTAIR, and more recently Jpx. The first applicaton of genome-wide RNA location analysis was by Chu et al. and Simon et al. (cited by the authors). They mapped roX2 binding genome-wide, and found that roX2 occupied Chromosome Entry Sites (CES) on X-chromosome; CES are known to recruit the dosage compensation machinery in trans when knocked into autosomes. Jpx site of action was recently found to be a specific CTCF site. The text should be revised to give due credit tor prior work and avoid hyperboles (this paper is perfectly fine without them).
We had not, of course, intended to overstate the novelty of this research and now have taken care to revise the manuscript including by removing the claim 'that this work is the first to study distantly located lncRNA binding sites'. We have expanded the Introduction citing further examples of trans-acting lncRNAs and have ensured that we now include a more detailed description of *roX2*, *Terc* and *Hotair* genomic binding on page 16 of the Discussion of the revised manuscript.

2. The authors were able to see effects with 52% reduction of Paupar level, which is a bit of a surprise. Is this related to the fact that Pax6 is haploinsufficient? The authors should at least comment on this unexpected sensitivity to Paupar level.
We also, at first, found it surprising that a 52% reduction in *Paupar* transcript levels had such a profound effect on gene expression. We believe that this is associated with the haploinsufficiency and dosage-sensitivity of *Pax6* (as mentioned in the Introduction). We now state in the Discussion (page 15) that "The widespread effect on over 900 genes when Paupar transcript levels were reduced by 52% may be associated with the haploinsufficiency and dosage-sensitivity of *Pax6*".

3. The broad Paupar binding sites can be up to 1 kb, which will contain many elements with complex and secondary effects. This should be discussed and at least acknowledged.
Agreed. We have since investigated further potential mechanisms of *Paupar* genomic targeting and have performed a refined motif analysis of *Paupar* CHART-Seq peaks. While we did not find enrichment of sequences complementary to *Paupar* within the *Paupar* Chart-Seq peaks (Methods, data not shown), we rediscovered the binding site motifs of several known transcription factors, including that of Pax6 (Supplementary Figure 5, Supplementary Table 7) enriched within *Paupar* CHART-Seq peaks. We suggest that *Paupar* is not targeted to the genome through direct RNA-DNA interactions but instead interacts with multiple different transcription factors in a context specific manner (page 10). In accordance with this, we show that Pax6 and *Paupar* co-occupy a specific subset of binding sites on the genome (Figure 6B).

4. The de-repression of reporter genes upon Paupar knockdown and the fact that Paupar binds to Pax6 suggests that Paupar may titrate Pax6 or another positive factor away from DNA. This would similar to the mechanism described for PANDA and it partner NFYA, Jpx for CTCF, and Lethe for NF-kB. If the authors can test this idea it may unify several observations in this paper.
We agree that this is an important experiment. Consequently, we performed ChIP-qPCR analysis at a set of *Paupar*-bound regions and showed that *Paupar* and Pax6 co-occupy binding sites within the

regulatory regions of genes whose expression are controlled by both *Pax6* and *Paupar* (Figure 6B). We then performed ChIP-qPCR to determine Pax6 occupancy at a number of these sites upon *Paupar* knockdown. This approach has been used previously to study the effect of PANDA knockdown on NFYA occupancy, RMST knockdown on Sox2 genomic binding and PRNCR1 and PCGEM1 knockdown on AR binding. Our results show that *Paupar* knockdown does not affect Pax6 chromatin residency (Supplemental Figure S7). In a similar manner, knockdown of the PRNCR1 and PCGEM1 lncRNAs does not affect binding of the AR to its target sites and instead PRNCR1 and PCGEM1 are involved in recruiting transcriptional co-factors to the AR (Yang et al, 2013).

Referee #2

Vance et al. examine the functional potential of the conserved lncRNA Paupar, expressed upstream of, and antisense to, the key neuro-developmental regulator gene Pax6. Employing shRNA-mediated 'knockdown' of Paupar coupled to microarray profiling, the authors suggest its involvement in gene expression in neural cells, either by up- or down-regulation of significantly enriched genes categorized by their ontology. The morphological phenotype of Paupar knockdown cells agrees with Paupar playing a role in controlling neural developmental programs. In addition, it appears that Paupar represses the expression of the associated Pax6 gene in a transcript level-dependent manner. Since Pax6 is a major neuro-developmental regulator, the possibility is considered that part of the Paupar transcriptional response may be indirectly via Pax6, thus, common and distinct transcriptional targets are dissected. Interestingly, Paupar also has some Pax6-independent transcriptional targets. Importantly, the recently established CHART-Seq technology is used to map binding sites of Paupar RNA genome wide, addressing the question whether also a long-distance trans-regulator function is present. Indeed, ~2850 genome-wide binding sites are identified preferentially within promoters and 5'UTRs.
We agree that our CHART-Seq results were important for elucidating *Paupar* function. The binding profiles for only a small number of lncRNAs have thus far been determined and not all of these have additionally intersected CHART-Seq results with expression profiling to identify bound and regulated genes.

Major concerns

1. In order to demonstrate a role of Paupar as a trans-regulator, the authors utilize luciferase reporter assays and show that a few cloned candidate binding sites of Paupar provide regulatory potential, which - in some cases - is also dependent on cellular levels of Paupar RNA. While implying a function of the respective Paupar binding sites, this analysis does not prove that the observed reporter regulation is exerted via direct binding of Paupar to the candidate regions. Since the stated novelty of this study is to verify a trans-acting role for a lncRNA, I think that more analysis is required at this point; e.g.
i) bioinformatics analysis of the Paupar binding sites aiming to identify any motif(s) that would support a significant 'recognition' by Paupar RNA (as also predicted by the demonstrated RNAseH sensitivity).
ii) mutagenesis of presented cases that would abrogate Paupar binding to DNA and consequently affect reporter regulation.
iii) in vitro validation of Paupar binding to identified DNA sequences (e.g. mobility shift assays).
In the interests of clarity, we would like to emphasise that CHART-Seq does not provide information about the nature of *Paupar*'s interaction with chromatin. This is because the protocol uses anti-sense oligonucleotides to purify target lncRNAs and their associated chromatin complexes, and the RNAseH elution step in the protocol digests RNA that is directly interacting with the anti-sense DNA probes. Both direct and indirect genomic associations are therefore identified using this approach. Targeting of *Paupar* could therefore be accomplished through direct base pairing with complementary DNA sequences, through RNA-RNA interactions at transcribed loci or indirectly through its association with sequence-specific DNA-binding transcription factors such as Pax6.
As suggested, we have now performed an indepth motif analysis of CHART-Seq peaks. We first used local alignment to search for sequences complementary to *Paupar* within the CHART-Seq peaks. This analysis (described in the Methods) did not discover an enrichment of *Paupar* complementary sequences compared to control size matched regions located upstream and downstream of the peaks. This suggested that *Paupar* is not targeted to the genome through direct

RNA-DNA interactions. We then performed separate motif discovery and enrichment analyses utilising the Meme-ChIP and AME algorithms. We discovered the presence of a motif closely resembling a known Pax6 binding motif in 9.2% of the top 500 peaks (Supplementary Figure 5C), and observed enrichments for binding motifs of several known neural transcription factors in *Paupar* bound sequences (Supplementary Table 7).
Together with our data showing that *Paupar* associates with Pax6 protein (Figure 6A) and that *Paupar* binding sites are enriched at genes regulated by both Pax6 and *Paupar* (Supplemental Figure S6), the presence of the Pax6 binding motif suggests that *Paupar* is recruited to a subset of its genomic targets through an interaction with the Pax6 transcription factor. Independently, we used ChIP-qPCR to test Pax6 occupancy at a set of *Paupar* binding sites and now show in Figure 6B that Pax6 and *Paupar* specifically co-occupy binding sites within the regulatory regions of genes whose expression change upon Pax6 and *Paupar* knockdown.

2. One always wonders how ncRNAs that are supposed to associate with chromatin to exert their function can be downregulated by shRNAs. Would it not be expected that primarily the nonfunctional cytoplasmic (and perhaps nucleoplasmic) pool(s) would be depleted? At a minimum the author should demonstrate that levels of chromatin-associated Paupar declines upon shRNA administration.
In response, we have now performed biochemical fractionation of N2A cells in which *Paupar* levels were depleted by transfection of a shRNA expression vector. We compared the relative amount of *Paupar* transcript in depleted cells to control cells transfected with a non-targeting shRNA (Supplemental Figure S2A). Results demonstrate that shRNA transfection can reduce the levels of chromatin associated *Paupar*.

3. What is the estimated copy number of Paupar per cell and is this compatible with the proposed function?
As suggested, we compared *Paupar* expression in a defined number of N2A cells to a standard curve of known *Paupar* copy number (generated by spiking RNA from ES cells, which do not express *Paupar,* with *in vitro* transcribed *Paupar* transcript). Our estimate is that *Paupar* is expressed at an average of 15 copies per N2A cell (Supplemental Figure S1B). As each CHART pull down is performed using approximately $8x10^7$ N2A cells these data indicate a stoichiometry of *Paupar* that is compatible with its proposed function as a *trans*-acting regulator of gene expression albeit differently in different cells.

Referee #3

The manuscript by Vance et al. describes the characterization of a long noncoding RNA termed Paupar, which is predominantly expressed in the brain. The authors contend that Paupar could regulate both its neighboring genes particularly pax6 and additional genomic sites through a trans-mediated mechanism.

Major comments:

1-If the authors contend that Paupar functions in trans as is suggested by the CHART-seq data; then they should show that

A) Paupar over-expression effects the expression of similar gene-expression program as the KD of pauper. Off course the down regulated genes should be upregulated and vice versa.
As suggested by the Reviewer, we performed *Paupar* over-expression experiments and used qRT-PCR to profile for expression changes in a set of candidate *Paupar* targets (Supplemental Figure S2C). Six of the eleven targets assayed yielded a clear dose-dependent effect upon *Paupar* overexpression. The ability of *Paupar* to induce gene expression changes when expressed from a plasmid therefore provides additional evidence that *Paupar* functions as a *trans*-acting transcriptional regulator.
Four of these genes (*Pax6*, *Sox1*, *E2f2*, *Cdc6*) were up-regulated upon *Paupar* knockdown and down-regulated following *Paupar* overexpression as would be expected, while a further two genes (*Suv39h1*, *Vamp1*) were down-regulated in both knockdown and overexpression experiments. It is far from surprising that *Paupar* overexpression did not induce expression changes in all genes tested and that the directionality of the expression changes between the knockdown and overexpression

experiments were not consistent in each case. An absence of expression change can be explained if *Paupar* is already bound to its genomic targets in N2A cells and if binding is saturated. Equivalent expression changes upon either knockdown or overexpression could be explained if *Paupar* overexpression has a dominant negative effect by titrating away a factor that is required for endogenous *Paupar* function. In addition, the required stoichiometry of co-factors may not be achieved for the over-expressed transcript to function effectively at all of its targets.

B) Paupar over-expression (perhaps they could remove the 20 or so nucleotide targeted by shRNA) should rescue the cell cycle defect (Figure 2D) and neurite outgrowth Figure 2F.
As our additional experiments (described above) revealed that *Paupar* overexpression does not in all cases result in reciprocal expression changes compared to the knockdown experiment we were concerned that overexpression experiments to rescue the cell cycle and neurite outgrowth effects would not necessarily provide clear and unambiguous findings.
A failure of over-expressed *Paupar* to rescue the knockdown effects would not necessarily imply that *Paupar* is unable to function in *trans* and could, instead, reflect a dependency on the precise levels of *Paupar* used in the assay. This would complicate the interpretation of the data. Furthermore, it would be difficult to precisely control the levels of over-expressed *Paupar* due to the presence of the shRNA expression construct in the knockdown cells. While the Reviewer suggests that we could remove the 20 nucleotides targeted by the shRNA, the functional domains of *Paupar* have not been mapped and such a deletion could result in uninterpretable functional changes.

C) They should perform structure/function analysis on Pauper determining the functional domains using expression analysis on Pax6 and other targets and whether the evolutionary conserved pieces are required for function.
We agree that such an analysis would further our understanding of *Paupar* function. However, the design of experiments mapping the transcriptional regulatory domains of *Paupar* would need to consider the gain or loss of binding to known protein interaction partners and/or *Paupar* genomic recruitment to further understand its mode of action. These experiments lie beyond the scope of the present study.

2- the overlap between the pauper KD gene expression changes by array and the bound sites using pauper CAHRT is quite poor. From 3170 sites about 242 are effected by Paupar KD. Is there a difference between the sites that are functionally affected and those that not. Are the sites affected have a higher level of gene expression; Are they up or down regulated upon Paupar knock-down. It is difficult to know whether Paupar is an activator or repressor of transcription.
To gain a more detailed understanding of *Paupar* function we examined the expression levels of *Paupar*-bound and -regulated genes. This analysis revealed that *Paupar* peaks are associated with genes that tend to be more highly expressed (Supplemental Figure S6). As suggested, we then hierarchically clustered the expression changes for *Paupar*-bound and -regulated genes. 202 out of 242 of these genes are down-regulated upon *Paupar* knockdown which indicates that *Paupar* functions as an activator at the majority of these sites (Figure 5F). The remaining 40 genes that are up-regulated upon *Paupar* knockdown suggest that *Paupar* can also function as a repressor at a subset of sites.
It is encouraging that the intersection between gene expression changes upon *Paupar* knockdown and *Paupar*-bound regulatory regions is comparable to those discovered in similar experiments for transcription factors that combine ChIP-Seq binding data with gene expression changes. In transcription factor ChIP experiments it has been shown that binding does not always correlate with function. For example, only a minority of regions bound by the key neuronal transcription factor REST are functional (Johnson et al, 2008). We also note that the gene expression changes profiled in our array experiments represent both direct and indirect effects and consequently we do not expect all regulated genes to be bound by *Paupar*.

3- The authors show that Pauper interacts with Pax6, does it effect Pax6 chromatin residence? They could assess this by KD and over-expression of Paupar at many of Pax6 tagtes.

In response, by first performing ChIP-qPCR experiments to measure Pax6 occupancy at a number of *Paupar* binding sites we identified genomic regions that are co-occupied by both *Paupar* and Pax6. These additional results are displayed in Figure 6B of the revised manuscript.
We then performed Pax6 ChIP experiments to determine Pax6 occupancy at a number of these sites upon *Paupar* knockdown. We used two independent targeting constructs in these experiments and

found that *Paupar* knockdown did not affect Pax6 chromatin residency (Supplemental Figure S7). We note that a recent study has shown that knockdown of the androgen receptor interacting PRNCR1 and PCGEM1 lncRNAs similarly had no effect on androgen receptor chromatin occupancy (Yang et al, 2013).

4- Frankly, it is difficult to interpret figure 6. Is there anything different between E2F2 site and E2F7 that result in differences in expression; Are there complementary sequences to Paupar in either of the putative Paupar binding sites. While the authors show that the E2F2BS site act as an activator (Figure 6A), Paupar KD result in increased expression Figure 2B, suggesting that Paupar is supposedly keeping the enhancer silent, this is a new mechanism of enhancer regulation which needs much more experiments to correctly decipher. Moreover, KD of Paupar resulting in the changes in SOX2BS and Hes1BS are so very small. I suggest removing this figure and performing complementary experiments with endogenous binding sites assessing gene expression changes and changes in chromatin signatures.

The reporter assays whose results are shown in Figure 6 are important because they illustrate the ability of *Paupar* to function in a *trans*-acting manner. These experiments indicate that *Paupar* depletion induces changes in the transcriptional activity of its binding sites when located in an episomal plasmid in a transiently transfected reporter. We do not yet fully understand why some sites respond to *Paupar* depletion while others do not, yet these differences are likely to reflect contrasting mechanisms of transcriptional regulation by which lncRNAs function. Reviewer 1 states that *Paupar* binding sites "will contain many elements with complex and secondary effects", and we believe this appropriately summarises the complexity of this issue. Our future experiments will be specifically addressing this issue.

We do not interpret the resulting changes in the activity of the *Sox2* and *Hes1* BS reporters upon Paupar knockdown as being insubstantial. Insertion of each of these *Paupar* BSs into the reporter resulted in a large, approximately 50%, reduction of SV40 promoter activity. When investigating the effect of *Paupar* knockdown we therefore consider it important to reflect on the effect of *Paupar* knockdown in relation to the activity of the binding sites: in other words, how much of this 50% repression is abolished. Knockdown of *Paupar* reduces repressive activity of the *Sox2* BS by approximately 50% and the *Hes1* BS repressive activity by about 30%. These are substantial changes in the ability of these elements to function as repressors. Furthermore, the observed effect is clearly dependent on *Paupar* transcript levels. We agree that the ability of *Paupar* to restrict the activity of the *E2f2* enhancer is of considerable interest. Studies have shown that transcriptional enhancers are bound by both positively and negatively acting factors and that repressors play a role in restricting the overall transcriptional output (Arnosti & Kulkarni, 2005).

To further understand *Paupar* genomic targeting and mode of action we have now performed MEME motif analysis and used a local alignment approach to search for sequences complementary to *Paupar* within CHART-Seq peaks. This did not yield an enrichment of *Paupar* complementary sequences compared to control size matched regions located upstream and downstream of the peaks. This suggests that *Paupar* is not targeted to the genome through direct RNA-DNA interactions. Indeed, our motif enrichment findings are consistent with an indirect recruitment mechanism where *Paupar* is targeted to the genome through several sequence specific DNA binding protein intermediates including Pax6 (see above). In accordance with this, we now show in Figure 6B that Pax6 and *Paupar* co-occupy binding sites within the regulatory regions of genes whose expression change upon Pax6 and *Paupar* knockdown. In combination with the findings that *Paupar* and Pax6 physically interact and that *Paupar* knockdown does not affect Pax6 genomic occupancy the data are consistent with a model in which Pax6 assists in the genomic targeting of *Paupar*.

References

Arnosti DN, Kulkarni MM (2005) Transcriptional enhancers: Intelligent enhanceosomes or flexible billboards? *J Cell Biochem* **94:** 890-898

Johnson R, Teh CH, Kunarso G, Wong KY, Srinivasan G, Cooper ML, Volta M, Chan SS, Lipovich L, Pollard SM, Karuturi RK, Wei CL, Buckley NJ, Stanton LW (2008) REST regulates distinct transcriptional networks in embryonic and neural stem cells. *PLoS Biol* **6:** e256

Yang L, Lin C, Jin C, Yang JC, Tanasa B, Li W, Merkurjev D, Ohgi KA, Meng D, Zhang J, Evans CP, Rosenfeld MG (2013) lncRNA-dependent mechanisms of androgen-receptor-regulated gene activation programs. *Nature* **500:** 598-602

2nd Editorial Decision 15 November 2013

Thank you for submitting the revised version of your manuscript to our editorial office; it has now been seen by one of the original referees whose comments are shown below.

As you will see, this referee finds that the experimental data provided in the revised manuscript is sufficient to address the major concerns raised for the original manuscript. However, the referee does ask you to carefully revise the discussion to more clearly acknowledge the remaining open questions regarding the mechanism of action for Paupar.

Thank you again for giving us the chance to consider your manuscript for The EMBO Journal, I look forward to your revision.

REFEREE REPORT

Referee #2

In the submitted revision, Vance et al. have addressed most of the reviewers' concerns. Given the somewhat unclear mechanism of Paupar function, I suggest the authors clarify their model/thoughts as much, and as precise, as possible in a revised Discussion. In doing so, the paper should make an interesting contribution to the lncRNA literature.

2nd Revision - authors' response 21 November 2013

We now submit a modified version of the manuscript in which we have carefully revised the discussion as suggested and have added a paragraph describing our model for *Paupar* mode of action. In it we state that ‘Our data are consistent with a model in which *Paupar* is indirectly targeted to the genome through RNA-protein interactions with multiple different neural transcription factors including PAX6. In accordance with this, we discovered a motif resembling a known PAX6 DNA binding motif within approximately 9% of the 500 top-scoring *Paupar* bound sequences and showed that *Paupar* and PAX6 co-occupy specific genomic sites within the regulatory regions of genes whose expression change upon both *Pax6* and *Paupar* knockdown. Furthermore, our data show that *Paupar* does not affect PAX6 chromatin occupancy and suggest that *Paupar* may regulate the association of PAX6 with its transcriptional cofactors to control target gene expression’.
